# A suite of macrocyclic peptide inhibitors and substrate probes for arginine methyltransferases

**DOI:** 10.1039/d5sc09232a

**Published:** 2026-02-25

**Authors:** R. Yoshisada, Y. Zhang, E. Janssen, C. Bouchard, D. A. Poole, T. Wan, L. R. Soares, I. M. Houtkamp, S. Abeln, H. Mouhib, M. J. van Haren, N. Marechal, N. Troffer-Charlier, V. Cura, J. Cavarelli, H. van Ingen, U. M. Bauer, N. I. Martin, S. A. K. Jongkees

**Affiliations:** a Department of Chemistry and Pharmaceutical Sciences, Amsterdam Institute of Molecular and Life Sciences (AIMMS), Vrije Universiteit Amsterdam De Boelelaan 1108 1081 HV Amsterdam The Netherlands s.a.k.jongkees@vu.nl; b Division of Chemical Biology and Drug Discovery, Utrecht Institute for Pharmaceutical Sciences (UIPS), Department of Pharmaceutical Sciences, Utrecht University Universiteitsweg 99 3584 CG Utrecht The Netherlands; c Biological Chemistry Group, Institute of Biology Leiden, Leiden University Sylviusweg 72 2333 BE Leiden The Netherlands; d Institute for Molecular Biology and Tumor Research (IMT), Philipps-University Marburg Hans-Meerwein-Str. 2 35043 Marburg Germany; e Department of Computer Science, Bioinformatics, Amsterdam Institute of Molecular and Life Sciences (AIMMS), Vrije Universiteit Amsterdam De Boelelaan 1105 1081 HV Amsterdam The Netherlands; f Department of Integrated Structural Biology, Institut de Génétique et de Biologie Moléculaire et Cellulaire, CNRS UMR 7104, INSERM U 1258, Université de Strasbourg Illkirch F-67404 France; g NMR Spectroscopy Research Group, Bijvoet Center for Biomolecular Research, Science Faculty, Utrecht University 3512 Utrecht The Netherlands

## Abstract

Arginine methyltransferases (PRMTs) are key regulators of chromatin structure, RNA processing, and signal transduction, and their dysregulation has been linked to cancer and other diseases. The development of potent and selective chemical probes for individual PRMTs remains a major challenge. Here we report a discovery campaign using mRNA display under a reprogrammed genetic code that yielded new macrocyclic peptide inhibitors and substrate probes for coactivator-associated arginine methyltransferase 1 (CARM1/PRMT4) and related family members. To fully exploit the sequencing data from these selections, we were necessitated to develop and implement a workflow that analyses complete datasets without arbitrary abundance cut-offs, based on rapid sequence clustering for redundancy reduction and followed by alignment to retain representative diversity for evolutionary analysis. Whereas conventional abundance-based analysis identified a dominant but weakly active sequence family, our comprehensive approach uncovered potent PRMT4-selective inhibitors, broader PRMT-active peptides, and efficient substrate sequences. This unexpected recovery of efficient substrates prompted structural investigation by NMR and molecular dynamics, which revealed distinct binding modes, including interactions outside the canonical substrate-binding cleft and conformational rearrangements upon binding. Overall, these results provide a new set of chemical biology tools for studying arginine methyltransferases and illustrate how full-dataset analysis can expand the diversity of hits from genetically encoded library discovery. With the growing prominence of mRNA display in both academic and industrial settings, this work highlights its value for identifying bioactive macrocycles with diverse functional profiles.

## Abstract 2

A broad panel of substrate and inhibitor peptides, with varied subtype selectivity, were identified by mRNA display against cofactor-associated arginine methyltransferase 1 (CARM1), enabled by efficient data processing of the full sequencing output.

## Graphical abstract

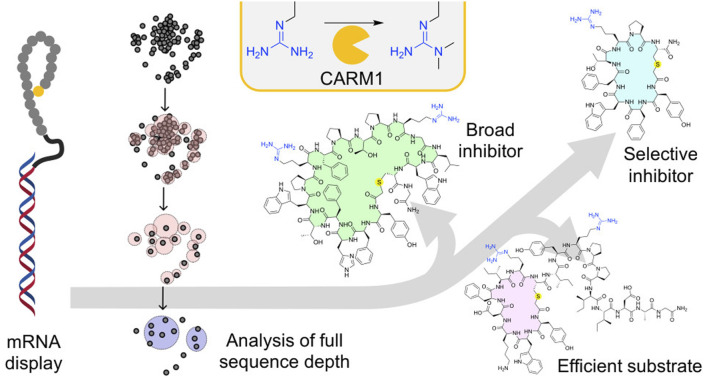

## Introduction

**Fig. 1:**
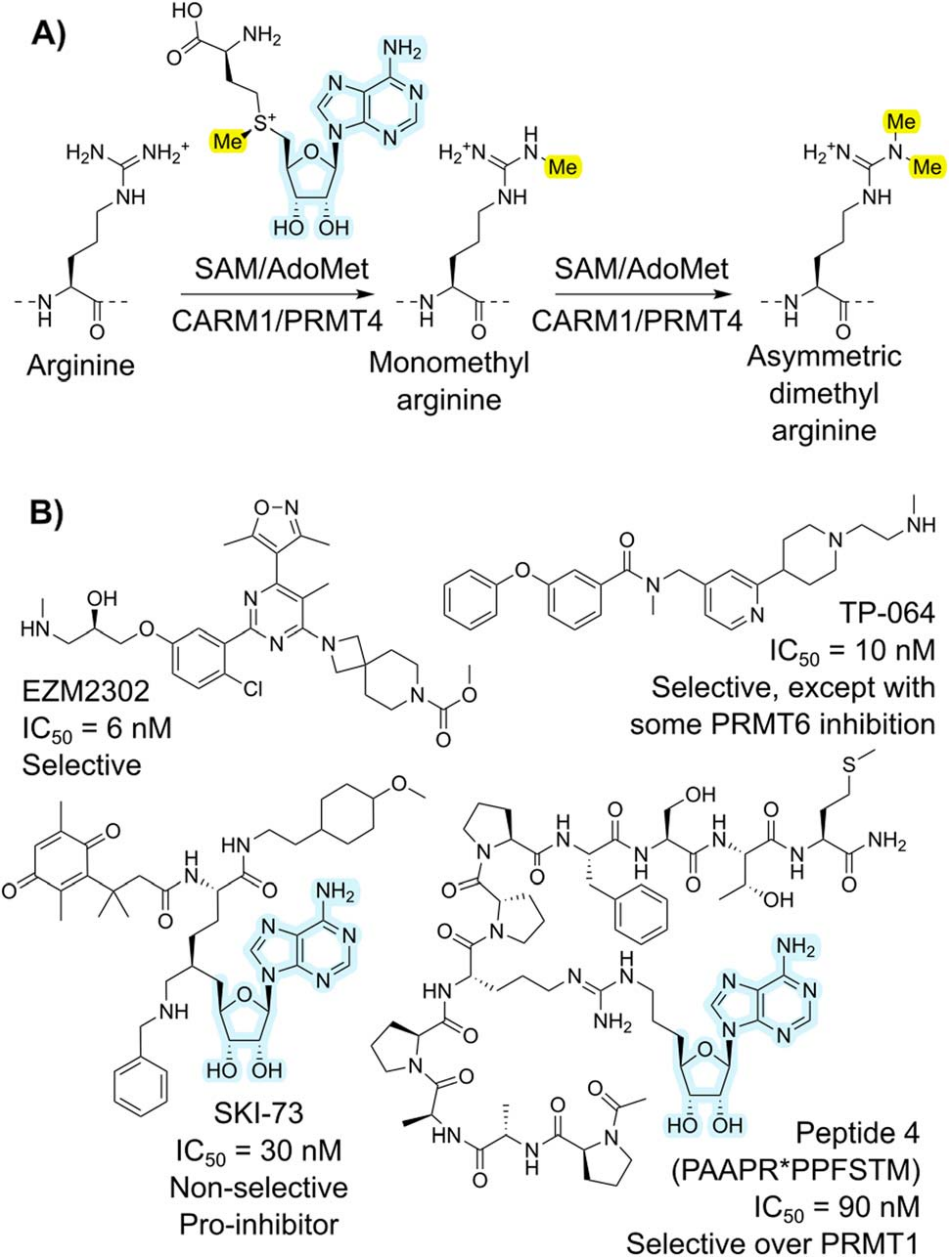
(A) Overall reaction catalyzed by CARM1, showing asymmetric dimethylation of arginine. Highlighted are the transferred methyl group in yellow and the adenosine cofactor fragment present in several inhibitors in light blue. (B) Example previously reported inhibitors for CARM1 across different compound types.

Coactivator-associated arginine methyltransferase 1 (CARM1), also known as protein arginine methyltransferase 4 (PRMT4), is a type I arginine methyltransferase that uses the *S*-adenosyl methionine (SAM) cofactor (Fig. 1A).^1,2^ This enzyme is part of a broader family of PRMT enzymes, subdivided into those that carry out asymmetric (PRMT1, PRMT2, PRMT3, PRMT4, PRMT6, and PRMT8; type I) or symmetric (PRMT5 and PRMT7; type II) methylation.^3^ These enzymes have broad substrate specificities and tend to engage substrate through backbone rather than sidechain interactions.^4^ Substrates of CARM1 methylation include histones (*e.g.*, H3), transcription factors (*e.g.*, p300), co-regulators of transcription (*e.g.*, CBP), splicing factors (*e.g.*, CA150),^5^ metabolic regulators (*e.g.* PKM2),^6^ and redox homeostasis components (*e.g.* Nrf2 and malate dehydrogenase 1).^7,8^ Malfunction or overexpression of CARM1 leads to critical defects, as shown by both knockout mice and enzyme-inactive CARM1 knock-in mice, which exhibit lethality.^9,10^ CARM1 overexpression has also been observed in several cancer types.^11–13^ Inhibitors of CARM1 are thus of interest in dissecting its biological roles, together with and distinct from other PRMT enzymes, and also have potential for translation to antitumor drugs. In general, the most potent PRMT inhibitors tend to focus on the relatively deep and strongly binding cofactor binding site, but the high conservation of this site means that such compounds struggle to achieve the selectivity that would allow dissection of this complex biology.^14^ Efforts to develop CARM1 regulators are ongoing.^1^ Example small molecules include EZM2302 (ref. 15 and 16) and TP-064 (ref. 17) (Fig. 1B), which occupy the substrate-binding pocket of CARM1 and have shown antitumor effects against cancers such as breast cancer, multiple myeloma (MM), acute myeloid leukemia (AML), and diffuse large B-cell lymphoma. In contrast, SKI-73 (ref. 18) inhibits CARM1 by blocking the *S*-adenosyl methionine (SAM) binding site.

Peptides are particularly appealing in this space because of their potential for high potency and selectivity, while also being less cytotoxic than small molecules on average.^19,20^ However, peptides inhibitors face challenges in biological stability and cell permeability.^21^ With proper design and development, for example macrocyclization and subsequent structural modification, modified peptides have been approved for clinical use.^19–21^ Because CARM1 is related to arginine methylation, it is perhaps surprising that few studies have reported peptide inhibitors for it. In one example, a peptide-SAM conjugate (Fig. 1B) was shown to act as a specific inhibitor of CARM1, where the peptide originated from a fragment of poly(A)-binding protein 1 (PABP1).^22^ The best of these compounds proved to be double-digit nanomolar IC_50_ inhibitors of CARM1/PRMT4, with over 100-fold selectivity over PRMT1, indicating the potential of peptide inhibitors. However, while these have value as structural probes for elucidating details of the catalytic mechanism, they are linear peptides that consequently have poor translation potential. Genetically encoded peptide libraries are receiving increasing attention as an alternative hit-finding method to such rational design approaches. Within these, mRNA display is a particularly high-throughput platform. This approach works by covalently connecting translated peptides (phenotype) to their encoding mRNA (genotype) *via* the antibiotic puromycin.^23,24^ Using flexizymes (flexible tRNA-aminoacylating ribozymes)^25^ or other tRNA-aminoacylation approaches,^26^ non-standard amino acids can be incorporated in peptides to adjust the chemical space of these libraries, introducing valuable features such as macrocyclization.^27^ Once enriched for binding to a target, the genetic information is sequenced. In recent years, this has often been performed using next generation sequencing (NGS) platforms such as those offered by Illumina.^28^ Even though the success of the mRNA display and all downstream applications is heavily dependent on the analysis of these sequencing data, high-throughput sequencing datasets are challenging to efficiently explore.

Traditionally, peptide sequences have been analyzed based on abundance, with ‘top hits’ proceeding to further assays. However, these ‘top hits’ are often derived from one or two clusters of sequences with strong sequence similarity, with a few mutations from the reference sequence, implying that ‘top hits’ selection gives a limited pool of hits that does not reflect the true potential of the experimental data. To diversify data exploration, other strategies have been attempted, but these are typically computationally expensive and therefore require a dataset of limited size by imposing an arbitrary cutoff. This leads to an under-exploration of lower ranked hits that could potentially have superior properties. For example, in our previous work we explored multiple sequence alignment of a subset of the most abundant sequences, followed by phylogenetic tree construction,^29^ and the Kawamura group employed clustering of all sequences with three or more counts using pairwise alignments combined with single-linkage hierarchical clustering.^30^ In phage display, where similar sequence analysis is required, the Derda group introduced a significance threshold combined with volcano plotting to compare sequences from target selection with those from a naïve selection. However, the actual analysis was performed by similarity-based clustering of the top 150 most abundant sequences.^31–33^ In the current work we demonstrate the value of mining the entirety of the dataset. Where ‘top hits’ in a RaPID selection against CARM1 revealed only a single family of modest hits, exploration of the entire dataset yielded multiple families with differing inhibition properties including a sub-micromolar 8-mer macrocyclic peptide inhibitor with excellent selectivity, broadly active PRMT inhibitors, and efficient *de novo* substrate sequences. We envision being able to further generalize this work as a strategy for generation of precise inhibitors of other PRMTs through RaPID screening and subsequent analysis of the entire high-throughput sequencing dataset.

## Results and discussion

### mRNA display selection reveals a dominant sequence family with poor activity

**Fig. 2:**
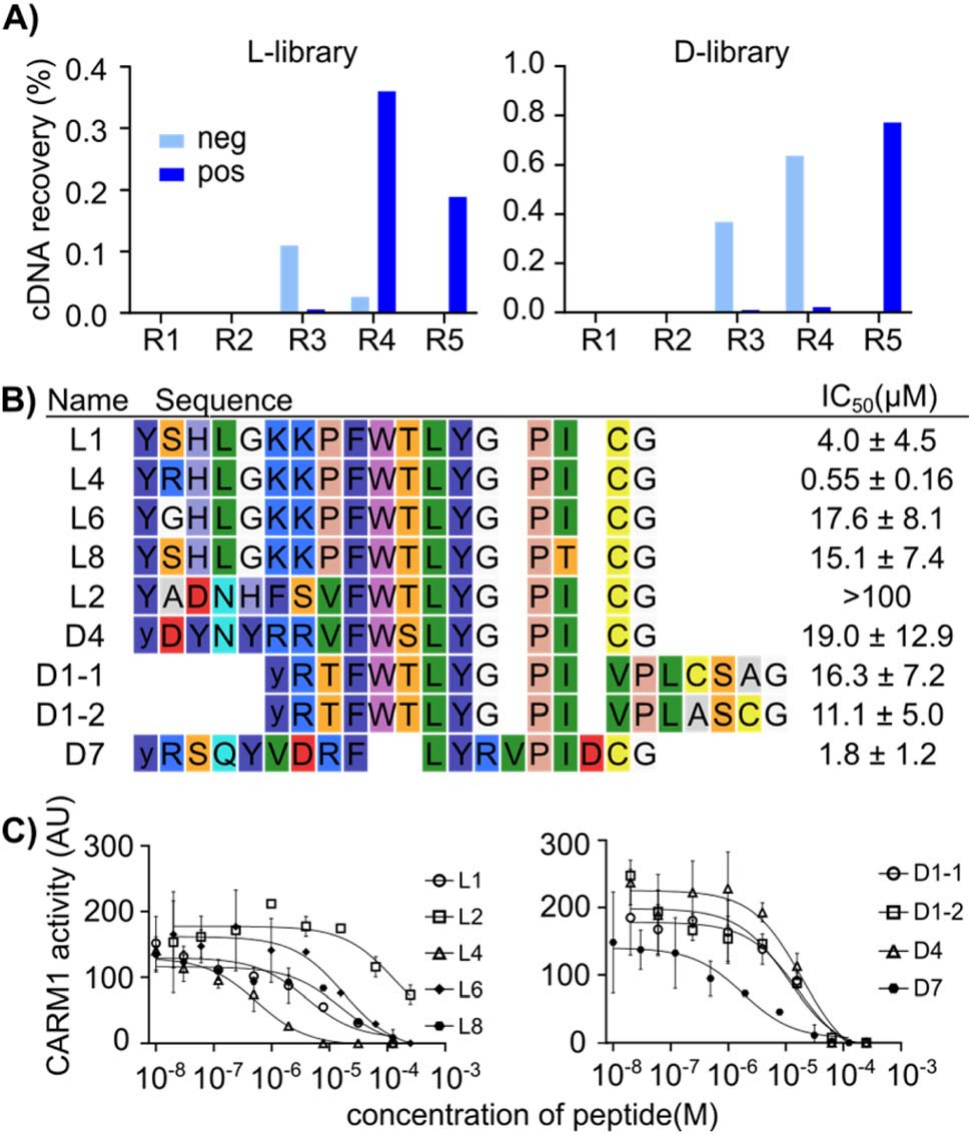
RaPID display selection using CARM1 immobilized on magnetic beads. (A) cDNA recovery over five rounds of the mRNA display selection. The l- and d-libraries differ only in the initiating amino acid, ClAc-l-Tyr-OH or ClAc-d-Tyr-OH, respectively. “neg” refers to cDNA recovered with streptavidin beads alone, while “pos” refers to cDNA recovered from biotinylated CARM1 immobilized on streptavidin beads. (B) Initial sequences picked *via* a single multiple sequence alignment of the 200 most abundant sequences. The chloroacetyl cyclization moiety is not shown, and lowercase “*y*” indicates d-Tyrosine. The IC_50_ values of the selected peptides are shown on the right. (C) Inhibition of CARM1 (286 nM) methylation of 12 µM PABP1^456−466^ peptide with 10 µM *S*-adenosyl methionine by varying concentrations of selected peptides, determined using a multiple reaction monitoring LC-MS assay.

A RaPID selection^27^ was performed to identify candidate peptide drugs and/or chemical biology tools targeting CARM1 using purified protein that was chemically biotinylated using NHS chemistry to allow immobilization on magnetic beads as a ‘bait’ for pull-down (Fig. S1A). The mRNA library followed a standard design that includes a ribosome-binding site, a peptide-encoding region with a start codon (AUG), a random region (15 NNK codons), a cysteine codon (UGC), and a flexible GSGSGS peptide spacer before a stop codon and a puromycin–oligonucleotide annealing region. As an initiating amino acid, chloroacetyl (ClAc)-l-Tyr or ClAc-d-Tyr were charged onto tRNA using flexizymes and employed in translation to allow for spontaneous cyclization with the cysteine encoded in the downstream region of the library. Two separate peptide libraries were prepared by *in vitro* translation of this mRNA using these two amino acids, referred to as ‘l’ and ‘d’ libraries from here on (reflecting the difference in initiating amino acid stereochemistry, with all other amino acids being of the canonical l-form). Following pulldown of peptide binders with the CARM1 bait protein, the iterative selection cycles were monitored by quantitative PCR of the output (compared with the input and non-selective binders in a negative selection without CARM1), and the selection was completed over five rounds (Fig. 2A) as indicated by a dramatic increase in recovery in rounds 4 and 5. Subsequently, the DNA pools recovered from each round were sequenced in high throughput (Illumina iSeq). Analysis of this dataset showed that the library was strongly enriched over successive rounds (Fig. S1B), converging on a clear preferred motif, as shown by the fact that the top 10 sequences occupied more than 40–50% of all reads in round 5 (Fig. S1C, S2 and S3). The top 200 most abundant unique sequences from both the l- and d-Tyr initiating libraries were analyzed by multiple sequence alignment, resulting in nine peptide sequences with a consensus motif (F-W-T-L-Y-G-P-I), along with one other sequence that was well enriched and did not contain this motif, that were selected to be characterized for their inhibitory potency using a multiple reaction monitoring (MRM) assay based on LC-MS determination of methylation of a substrate peptide derived from PABP1 (Fig. 2B and C).

All of these candidates exhibited modest inhibition, with the best being peptide L4 at an IC_50_ of 0.5 µM (MLM assay, 286 nM enzyme, 10 µM SAM substrate, 12 µM peptide substrate) and all others having IC_50_ values greater than 1 µM. Given that RaPID selections often result in sub-micromolar or even sub-nanomolar binders,^34^ we then assessed whether this low activity was due to poor binding or other factors, for example only partial overlap of hit binding with the active site. A fluorescence polarization assay with a FAM-derivative of peptide L1 showed a *K*_d_ of 132.7 nM against CARM1 (with 95% confidence between 106.3 to 168.5 nM) (Fig. S4).^53^ This value is in line with affinities of previously selected mRNA display hits, and so indicates that binding does not appear to be the cause of poor inhibition. This in turn may indicate mixed inhibition modes from poor overlap with functional sites.

### Analysis of the full dataset reveals previously hidden conserved sequence motifs

**Fig. 3:**
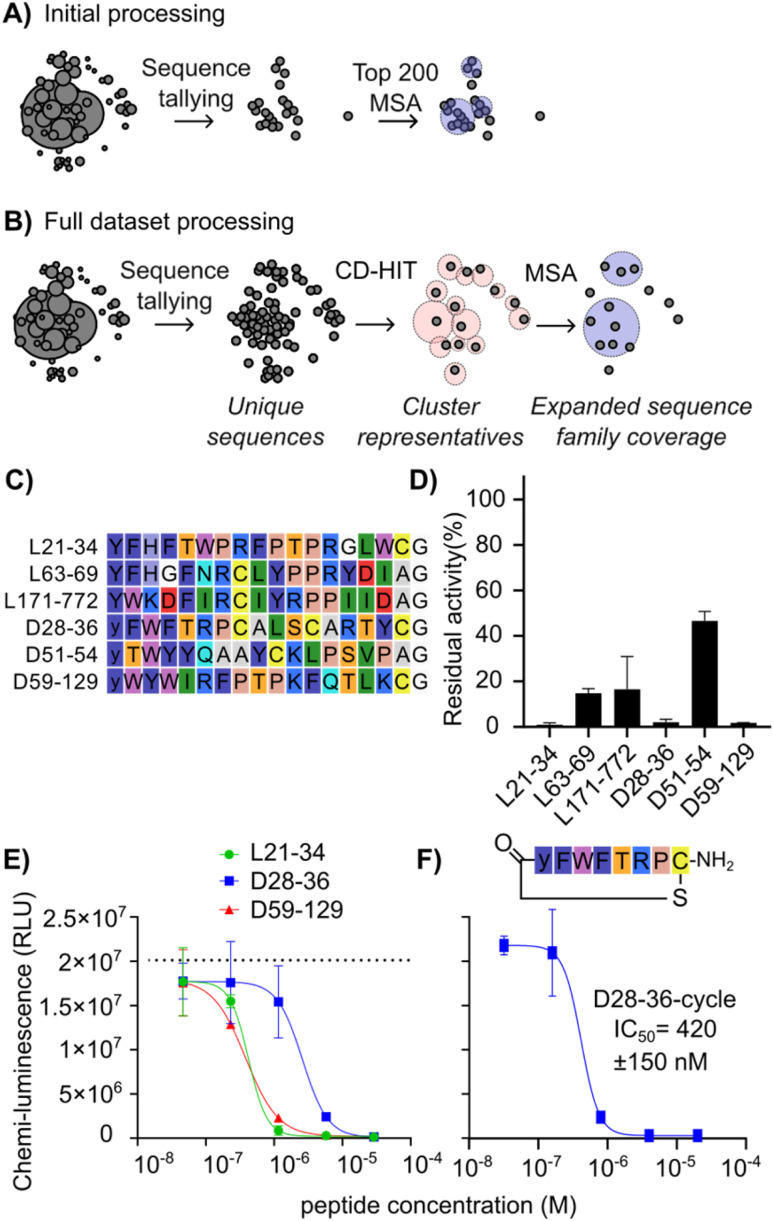
Illustrative comparison of sequence analysis workflows to improve the diversity and quality of hits picked for follow-up studies, and CARM1 inhibition by newly discovered peptides. (A) Sequence picking from the top 200 enriched sequences as used to select hits in Fig. 2. Circles represent unique sequences, and their size indicates the number of counts. Spatial separation reflects sequence identity/relationships. Lighter circles across multiple sequences represent sequence families with conserved motifs. (B) Sequence picking by redundancy reduction followed by MSA of the seed sequences as used to find additional hits. (C) List of peptides chosen as representatives of main clusters in the multiple sequence alignment following additional data processing. (D) CARM1 (245 nM) activity in the presence of tested peptides at 100 µM, shown as residual activity relative to no inhibitor. (E) CARM1 (245 nM) activity with varying concentrations of the three most potent peptides from (A), measured using a plate-based chemiluminescence assay with antibody detection of methylation. Note that the substrate peptide is plate-immobilized, and concentration is unknown. Dotted line indicates positive control without inhibition. (F) CARM1 activity with varying concentrations of D28-36-cycle, with conditions as for (E).

To search for further candidates for testing, we deployed a sequence analysis workflow that avoids the use of an artificial threshold for sequence counts (Fig. 3A and B). This workflow first uses CD-HIT (cluster database at high identity with tolerance) clustering^35–37^ as a first pass to provide ultrafast data reduction across the entire dataset by condensing near duplicates into representative sequences, followed by more computationally intensive multiple sequence alignment (MSA)^38,39^ of the representative sequences to refine clusters and allow for expert picking of promising candidates. The use of CD-HIT in clustering of NGS outputs has previously been used in phage display to remove redundancy from a training dataset of 80 seed sequences for machine learning.^40^ In this work we apply it with a higher similarity threshold to process the entire sequencing dataset in an efficient way with minimal change to the sequence families represented. This analysis was focused on round 3 (one round before strong enrichment was observed by qPCR) to maximize sequence diversity present.

This workflow resulted in a list of sequences from broader, previously hidden clusters (Fig. 3C). These new hits were named as “[library][cluster number] − [abundance rank out of all sequences from round 3]”—for instance, L21-34 indicates a sequence from the l-library, the 21st CD-HIT cluster, and the 34th most abundant sequence of all sequences. Sequences L21-34, L63-69, L171-772, D51-54, and D59-129 were not found in the top 200 sequences of the round 5 dataset due to the strong enrichment of the main motif, where there were few other sequences outside of the main family (Fig. S2 and S3). Although D28-36 was present in the initial analysis, it was considered an outlier since it was a single representative with no other homologues (Fig. S1 and S2, position D121). Other homologues were deeply hidden in the sequence pool, as seen in the sequence family of D28-36 (Fig. S5), making it unlikely to be selected as representing a real hit. This new set of potential hits (Fig. 3C) was tested for inhibition of CARM1 at a high initial concentration of 100 µM peptide. This was done to ensure the detection of weakly active sequences in this initial screen. This new set of assays was carried out using a commercial assay kit with a luciferin-conjugated antibody detecting methylated arginine in an undisclosed peptide substrate coated on the plate. This assay was chosen for its high throughput and to minimize consumption of limited protein stock.

As shown in Fig. 3D, peptides L21-34, D28-36 and D59-129 reduced PABP1^456−466^ methylation to less than 3%, followed by L63-69 (15%), L171-772 (27%), and D51-54 (47%). For the top three inhibiting peptides, a titration was performed (Fig. 3E) resulting in IC_50_ values of 260 ± 90 nM for D59-129, 290 ± 60 nM for L21-34, and 1.8 ± 0.7 µM for D28-36 (ELISA assay, 245 nM enzyme, 1 µM SAM substrate, unknown concentration of plate-bound substrate). Since D28-36 displayed a highly conserved cyclic motif and a poorly conserved tail in the alignment (Fig. S5), its macrocyclic component was also synthesized and tested separately. To our surprise, the inhibitory ability was not only maintained but appeared 4.3-fold stronger than that of the full sequence (D28-36-cycle IC_50_ = 420 ± 150 nM) (Fig. 3F). The depth of sequence information obtained from our strategy of alignment after complexity reduction was crucial here in revealing this lack of conservation in the tail region and thereby making clear where the peptide could be truncated. The small size of this truncated peptide as compared to the other hits (8-mer *vs.* 17-mer) means that it is better positioned for development into a cell-active compound. While it is not more potent than existing CARM1 inhibitors discussed in the Introduction, it represents an as-yet unoptimized structure and could potentially be enhanced by addition of non-canonical amino acids.

**Fig. 4:**
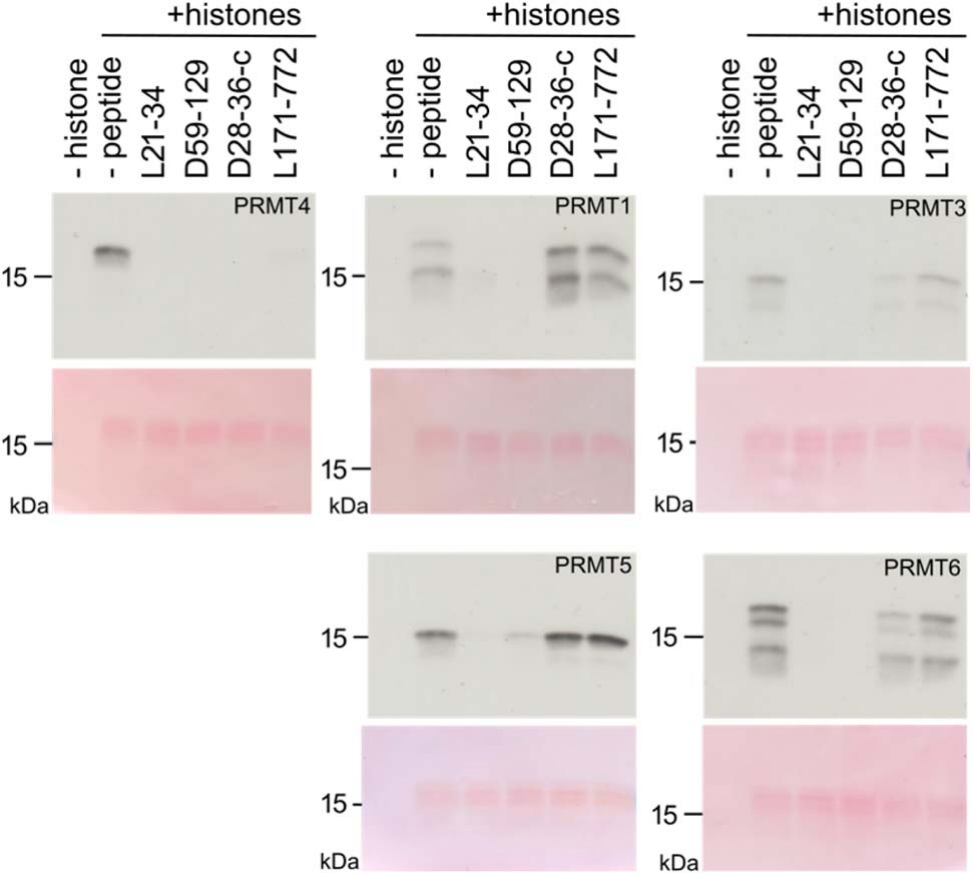
Specificity of test peptides. *In vitro* histone methylation assays with PRMT proteins (PRMT1, PRMT2, PRMT5, and PRMT6) in the absence (–peptide) or presence of test peptides (L21-34, D59-129, D28-36-cycle, and L171-772). For each pair, the upper panel displays the autoradiographic detection of methylation with [^14^C]-SAM, while the lower panel shows Ponceau Red staining as histone protein loading control.

We next tested the inhibition specificity of the discovered peptides across the PRMT family by testing histone methylation. Each peptide (L21-34, D59-129, D28-36-cycle, and L171-772) was incubated at 100 µM together with bulk histones as protein substrate and [^14^C-methyl]-*S*-adenosyl-methionine ([^14^C]-SAM) as cofactor to enable sensitive product detection. The peptides were tested against PRMT4 (=CARM1), PRMT1, PRMT3, PRMT5, and PRMT6. The samples were subjected to SDS-PAGE followed by blotting, and stained with Ponceau Red to monitor histone loading before autoradiographic detection (Fig. 4 and S6).

While all four macrocyclic peptides were most effective at inhibiting PRMT4, they had less (L21-34, D59-129) or no inhibitory (D28-36-c, L171-772) effect on PRMT5 enzymatic activity (Fig. 4 and S6), a type II PRMT that catalyzes symmetric di-methylation. Macrocyclic peptides L21-34 and D59-129 showed also strong inhibitory effects on other type I PRMTs (PRMT1, PRMT3, PRMT6) in this more physiological setting, indicating that they are broadly active inhibitors. In contrast, D28-36-cycle and L171-772 suppressed PRMT4 activity with high selectivity and revealed no or a small reciprocal effect on the catalytic activity of the other PRMTs. For example, L171-772 appeared to enhance the activity of PRMT1, while D28-36-c appeared to weakly enhance the activity of PRMT5. A serial dilution assay for these two peptides was performed, revealing a complete inhibition of PRMT4 by 20 µM D28-36-cycle and weaker inhibition by L171-772 (Fig. S7). This trend is consistent with the inhibition observed for inhibition of PABP1^456−466^ peptide methylation *in vitro* (Fig. 3D). All together, these inhibitors exhibit a range of selectivity profiles, from highly selective to broadly active across type I PRMTs, underscoring the value of testing different hits revealed by our new data analysis approach.

### Novel peptide substrates revealed by mRNA display

**Fig. 5:**
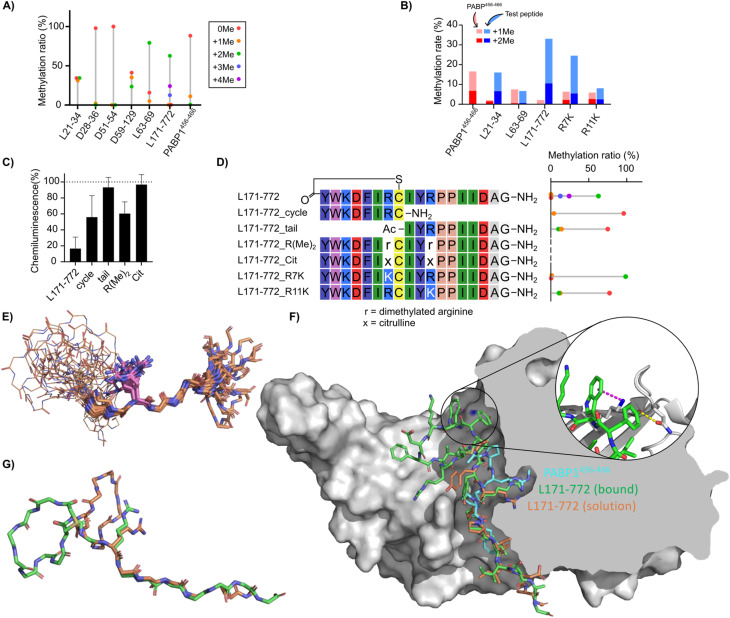
Novel peptide substrates identified by mRNA display. (A) Ratio of peptide methylation states following treatment of macryocyclic peptide inhibitors with CARM1 and SAM, in the presence and absence of additional peptide substrate, as determined by LC-MS. Relative abundance of methylated and non-methylated peptides (8.3 µM) after 18 hours of reaction with 300 nM CARM1 and 83 µM SAM are shown by bar height. (B) Competition reactions between the PABP1^456−466^ peptide (red) and each test peptide (blue) at 7.7 µM each. The summed percentages of monomethylated and dimethylated products are shown as stacked bars. Methylation of PABP1^456−466^ in the absence of competitor is shown on the left. (C) Fragments and variants of L171-772 tested for methylation shown as in (A) (rotated to align with sequence table). (D) CARM1 inhibition assay with L171-772 derivative peptides (100 µM) using chemiluminescent ELISA assay as in Fig. 3D. (E–G) Structural studies of the L171-772 peptide. (E) Superposition of 20 lowest energy conformations of L171-772 based on solution NMR constraints, aligned to the sequence motif IYRPPII in the tail region. Macrocycle backbones are shown as lines, tails as sticks, and with the sidechain of Arg11 depicted explicitly in magenta. (F) Bound pose of L171-772 (green) from molecular dynamics simulation with CARM1 (grey), overlaid with an arbitrary conformation of the NMR ensemble (orange) and the PABP1^456−466^ peptide from PDB 5DXA (cyan). Cutaway of the right half of the protein shows the arginine-binding pocket. (Inset) The cation–π interaction between L171-772 Trp2 and CARM1 Lys337 (blue surface) is shown as a magenta dotted line and the hydrogen bond between L171-772 Tyr1 and CARM1 Gln313 is shown as a yellow dotted line (G) overlay of backbones for the L171-772 bound state from molecular dynamics (green) with one conformation from the NMR ensemble (orange), showing the tail overlap and the different relative orientations of the macrocycle (Arg11 sidechains shown as stick representation).

All inhibitory macrocyclic peptides contained one or two arginine residues. Since CARM1 possesses an arginine-binding pocket and methylates the residue that binds in this pocket, we hypothesized that arginines in the peptides play a key role in the inhibition mechanism and could potentially themselves be methylated. We considered that substitution of this arginine with an analogue could increase inhibitory potency, or that efficient substrate peptides could be good candidates for a bisubstrate-inhibitor approach by connection of the arginine to a SAM-derived fragment. Therefore, we assessed whether the peptide inhibitors could also be methylated by measuring the ratio of the unmethylated peptide and methylated peptide using LC-MS (Fig. 5A),^41^ ensuring that peptide concentration is above that of the enzyme to allow for multiple turnover and so to also assess substrate binding and release. The known substrate PABP1^456−466^ showed 11% of mono- and 0.5% of di-methylated species in this assay. Of our new peptides, L21-34 produced 31% of mono- and 34% of di-methylated species, D59-129 gave 35% of mono- and 24% of di-methylated species, and L63-69 yielded 5% of mono- and 79% of di-methylated species. The best substrate, L171-772 produced 0.2% of mono-, 63% of di-methylated, as well as 13% of tri- and 24% of tetra-methylated products, indicating that two arginines in the L171-772 analogue were each methylated twice (of note, this was not the case in the somewhat similar sequence L63-69). All of these methylation rates were higher than those observed with the control substrate PABP1^456−466^. In contrast, D51-54 showed no methylated species, consistent with the lack of arginines and its weak inhibitory activity. For D28-36, despite the presence of an Arg–Pro motif known to be present in several natural substrates,^42,43^ the observed methylation was only 2.1%. The majority of these peptides were therefore confirmed to be engaging the active site as substrates.

We were surprised to recover from the mRNA display sequences that seemed to be more efficiently methylated than canonical peptides derived from a native protein substrate. Enzyme catalysis requires both binding and release of substrate, which seems at odds with the stringent washing steps of our selection method, which tends to favor slow off-rates. Although finding new substrate sequences was not necessarily our goal, we were curious to compare the apparent relative rates of methylation of our three most efficient substrates (L21-34, L63-69, and L171-772) with that of the known substrate PABP1^456−466^. The newly identified peptide substrates were mixed in an equimolar ratio with the canonical substrate and allowed to compete for methylation, as a direct measure of relative efficiency without requiring full kinetic characterization (again due to limiting amounts of enzyme). A control experiment was performed by replacing the test peptide with ultrapure water. In all three competition assays (Fig. 5B), PABP1^456−466^ methylation was suppressed. Specifically, in the competition between L171-772 and PABP1^456−466^, 23% of mono- and 10% of di-methylated L171-772 were detected, while only 2% of PABP1^456−466^ was monomethylated and no dimethylation was observed. L171-772 peptides with more than three methylations were not detected under these conditions. For L21-34, 9.4% of mono- and 6.9% of di-methylated product were found, whereas 0.4% of mono- and 1.6% of di-methylated PABP1^456−466^ were detected. These results indicate a strong inhibition of PABP1^456−466^ methylation by L171-772 and L21-34 through competition for enzyme turnover. In the competition between L63-69 and PABP1^456−466^, 6.1% of mono- and 0.6% of di-methylated L63-69 products were detected, whereas 7% of mono- and 0.5% of di-methylated PABP1^456−466^ products were found, indicating a weaker inhibition by L63-69 compared to the other two peptides. To attempt to convert the substrate peptide L171-772 into a simple inhibitor, we synthesized two peptides with both of the arginines replaced with either asymmetrically di-methylated arginine or citrulline. Inhibition by 100 µM tetra-methylated peptide was only around 60% while the peptide containing citrulline was found to be completely inactive as inhibitor (Fig. 5C). The lack of inhibition by citrullinated peptide likely reflects the important contribution to binding from the positive charge, while the poor inhibition by the tetramethylated peptide likely results from a strategy by the enzyme to limit product inhibition. CARM1 has been shown to bind effectively to non-methylated or mono-methylated arginines, but much more poorly (by 3 orders of magnitude) to di-methylated product.^44^ Given the poor binding of methylated products, it seems likely that the L171-772 peptide only enriched because of a lack of turnover in the display experiment, as no excess of cofactor was added to the RaPID selection.

The substrate properties of the most active lariat peptide, L-171-772, were next examined in more detail by dissecting it into its cyclic (L171-772_cycle) and tail (L171-772_tail) fragments, each containing one arginine that can be methylated. Each fragment was synthesized, and its methylation activity was assessed by LC-MS (Fig. 5D). After overnight reaction, a mere 4% of mono-methylated L171-772_cycle was found and no di-methylated products were detected, while 14% of mono- and 10% of di-methylated L171-772_tail products were detected (and inhibition by each fragment was also decreased relative to the parent, Fig. 5C). This indicates that the tail fragment has a stronger methylation capacity but the methylation capacity of both fragments was strongly reduced, suggesting that the conformation of the peptide is important for methylation and is not conserved in the two fragments. To further validate the methylation capacity of each arginine in the context of a full-length peptide, L171-772_R7K and L171-772_R11K peptides were synthesized and their methylation capacity was tested (Fig. 5D). Here, arginine at position 7 or 11 was replaced with lysine, as a minimal mutation that conserves charge but cannot be methylated. While arginine on position 11 (R7K) showed almost full di-methylation (>98%), the arginine in position 7 (R11K) showed only 12% of mono- and 11% of di-methylated product. Competition methylation experiments with these arginine-to-lysine mutated L171-772 peptides and PABP1^456−466^ (Fig. 5B) showed that 6.3% of PABP1^456−466^ was mono- or di-methylated in the presence of L171-772_R7K, and 8.1% in the presence of L171-772_R11K. Since 17% methylated product was detected without any competitor, both mutants were found to be inhibitory, with a slight advantage for the tail arginine (and so L171-772_R11K still showed more than 50% inhibition). These results confirm that the tail arginine is a stronger methylation substrate, while also indicating that the cyclic region contributes to this reactivity and that the peptide analogue can still bind to the pocket even without a strong methylation substrate activity, and that inhibition thus arises not only from competition for activity.

To investigate the underlying basis for the differences in methylation ability between these two Arg residues in L171-772, as well as to attempt to explain the unexpectedly high methylation-accepting ability of Arg11 in particular, we solved an NMR structure of the peptide and modelled its interactions with the substrate binding site of CARM1 by molecular dynamics. The NMR structure of the free peptide in solution (Fig. 5E) showed a well-defined extended conformation of the tail, likely driven by the multiple proline residues, while the macrocycle is compact and relatively well conserved. However, the spatial relationship between the macrocycle and the tail is highly variable. Notably, the structural context of Arg11 in the tail is very well conserved. The free state of peptide was compared with a bound-state model derived by molecular dynamics. In this, the highest-scoring bound pose had the hydrophobic residues in the macrocycle buried and hydrophilic/charged residues exposed to solution, with roughly equal binding contributions from the macrocycle and the tail (−32.0 and −32.5 kcal mol^−1^ obtained from MD). Notable interactions from the macrocycle include a hydrogen bond between CARM1-Gln313 and L171-772-Tyr1 (2.9 Å) and a cation–pi interaction between CARM1-Lys337 and L171-772-Trp2 (3.9 Å, 89° lysine-NH_2_ to ring center, Fig. 5F). The model of the bound state retained the same extended tail conformation as the NMR structure, but the loop orientation changed dramatically to avoid steric clash with the protein (Fig. 5G). The tail of L171-772 in the bound state model overlaps well with the NMR structure (RMSD 1.855 Å across the IYRPPII fragment), and even more so with the co-crystal structure^45^ of PABP1^456−466^ (RMSD 0.149 Å across the IYRPPII fragment). The L171-772 peptide solution conformation therefore appears to be well primed to bind CARM1 as a substrate, and the additional buried hydrophobic surface of the macrocycle provides a rationale for why the full lariat structure is a much better substrate than the tail alone. These additional interactions created by the macrocycle also provide a rationale for why this compound is a better substrate than the protein-derived peptide fragment PABP1^456−466^.

## Conclusion

We report here the discovery of a suite of macrocyclic peptide inhibitors of CARM1/PRMT4 by mRNA display under a reprogrammed genetic code. In this selection campaign, standard data-processing methods by a sequence abundance cut-off followed by multiple sequence alignment on the final most enriched round of selection resulted in a single dominant sequence family, which proved to be poorly active. Re-analysis of the same sequencing output using an approach that allowed analysis of the full dataset in an earlier round revealed numerous additional clusters, including peptides L21-34, D59-129 and D28-36, which inhibit PRMT4 through active site engagement. The cyclic fragment of peptide D28-36 (D28-36-cycle) showed over fourfold stronger inhibition than the lariat parental sequence and was highly specific for PRMT4 over other PRMTs. Complementary to this, peptides L21-34 and D59-129 exhibited a broad cross-inhibition of the type I methyltransferases PRMT1, PRMT3, PRMT4, and PRMT6 and to a lesser extent of the type II methyltransferase PRMT5. These peptides are therefore highly promising tools for future dissection of PRMT signaling. In addition, several sequences proved to be efficient substrates for PRMT4 methylation. Selection by mRNA display involves stringent washing that generally selects for tight binding and slow off-rates. These characteristics are typically incompatible with efficient catalysis, which requires ligand dissociation to allow new substrate binding. Enrichment of substrate sequences appears to have been possible in this case because the selection was carried out in absence of cofactor, preventing the di-methylation that normally triggers product release. This suggests that selection in the presence of cofactor, or with different post-translational modifications,^46^ might lead to further distinct hits. In future work development will focus on the D28-36 macrocycle, which shows a high potential for cellular activity, as well as on taking lessons from L171-772 to leverage its high efficiency as a substrate to allow improved design of bifunctional inhibitors containing a fusion of peptide and *S*-adenosyl methionine.^47^ In addition, selections against other biologically important arginine methyltransferases, such as PRMT1, could generate an arginine methyltransferase toolbox for chemical biology, as has recently been reported for an arginine deiminase.^48^ Our results emphasize the need to carefully consider which dataset to process from an enrichment trajectory using a genetically encoded library, as well as the importance of computationally efficient algorithms that can process the entirety of the dataset.

## Author contributions

R. Yoshisada: conceptualisation, methodology, software, formal analysis, investigation, writing – original draft, visualization. Y. Zhang: methodology, investigation. E. Janssen: methodology, investigation, writing – review & editing. C. Bouchard: methodology, investigation, formal analysis, writing – review & editing. D. A. Poole III: methodology, investigation, writing – original draft. T. Wan: investigation. L. R. Soares: investigation. I. M. Houtkamp: methodology, software. S. Abeln: supervision. H. Mouhib: supervision. M. J. van Haren: supervision. N. Marechal: resources. N. Troffer-Charlier: resources. V. Cura: resources. J. Cavarelli: resources, funding acquisition. H. van Ingen: investigation, writing – review & editing. U. M. Bauer: supervision, funding acquisition. N. I. Martin: conceptualisation, supervision. S. A. K. Jongkees: conceptualisation, writing – review & editing, visualization, supervision, project administration, funding acquisition.

## Conflicts of interest

There are no conflicts to delcare.

## Supplementary Material

SC-017-D5SC09232A-s001

## Data Availability

All other data are included in the manuscript supporting information (SI). Supplementary information: the authors have cited additional references within the supporting information.^49–71^ Raw sequencing data are available from DOI: 10.34894/Q4HR8A while https://github.com/yoshisadades/CARM1_NGS_analysis contains data processing scripts. See DOI: https://doi.org/10.1039/d5sc09232a.
