# Supplementary material for: A suite of macrocyclic peptide inhibitors and substrate probes for arginine methyltransferases
Source: Chem Sci. 2026 Feb 25;17(16):7986–96. doi: 10.1039/d5sc09232a (PMC12958360; doi:10.1039/d5sc09232a)
Supplement: SC-017-D5SC09232A-s001 [file SC-017-D5SC09232A-s001.pdf]

Supplementary Information (SI) for Chemical Science.
This journal is © The Royal Society of Chemistry 2026

Supporting information

Supplementary Figures

Figure S1. Cumulative percentage of top 100 abundant sequences in the sequencing output. 2
Figure S2. Multiple sequence alignment of top 200 abundant D-library originating sequences from round 1 to round 5. 3
Figure S3. Multiple sequence alignment of top 200 abundant L-library originating sequences from round 1 to round 5. 4
Figure S4. Binding of peptide L1 by fluorescence polarization 5
Figure S5. Sequence family with D28-36 as founding member 5
Figure S6. *In vitro* histone methylation assay with PRMT1, PRMT3, PRMT4, PRMT5, PRMT6 in the presence of test peptides. 5
Figure S7. *In vitro* methylation assay with PRMT4 in the presence of D28-36-cycle and L171-772 at varied concentrations. 6
Figure S8. PRMT4 chemiluminescent assay with a control peptide PABP1$^{456-466}$. 10

Materials and methods

Materials. 6
mRNA display selection. 7
Solid Phase peptide Synthesis. 7
Fluorescent Polarization. 9
CARM1 inhibition assays. 9
In vitro histone methylation assays with PRMTs. 11
Structural work (NMR and MD). 11

Supplementary Tables

Table S1. NMR peak table. 13
Table S2. Restraints for structural refinement. 17
Table S3. Derived angle restraints from TALOS+. 21
Table S4. Structural statistics for L171-772. 21

Spectra 22

References 46

A

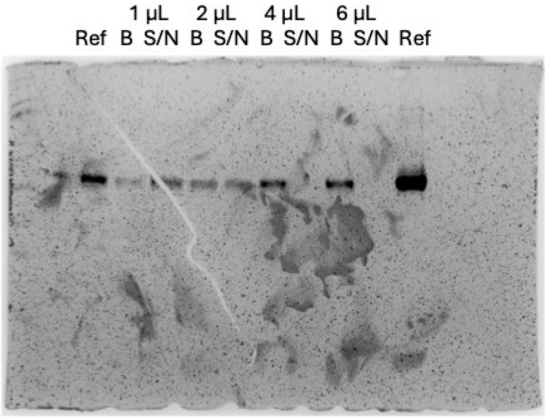

B

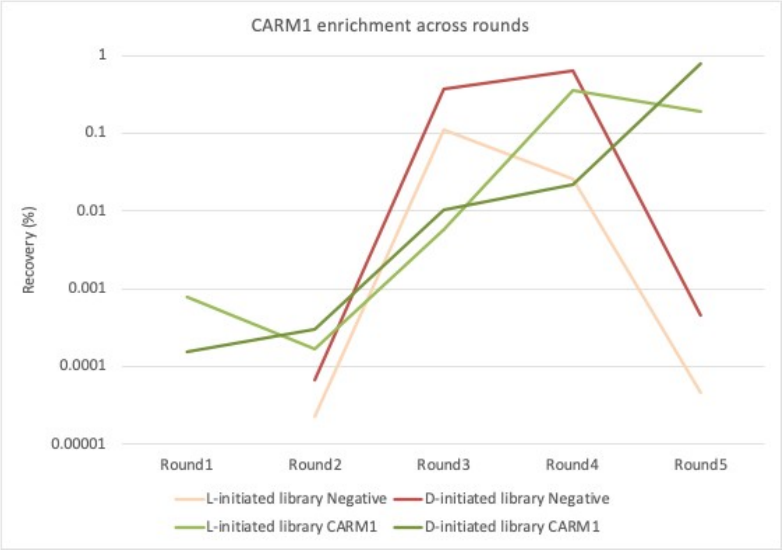

C

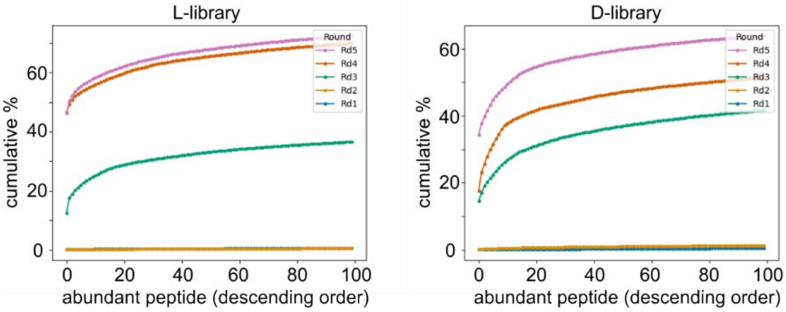

Figure S1. A) Protein immobilization on streptavidin magnetic beads (volumes as indicated), loading 200 ng protein in each pair of lanes (S/N, supernatant; B, beads; Ref, reference band). B) Recovery of library after each round of selection, as determined by qPCR of input and output. Negative refers to recovery with beads without target protein (last of 3 or 7 repeats, not carried out in round 1). C) Cumulative percentage of top 100 most abundant sequences in the sequencing output, plotted for each round. Round 3, representing the first clear enrichment of target-binding sequences, was thus taken for analysis by CD-HIT and MSA.

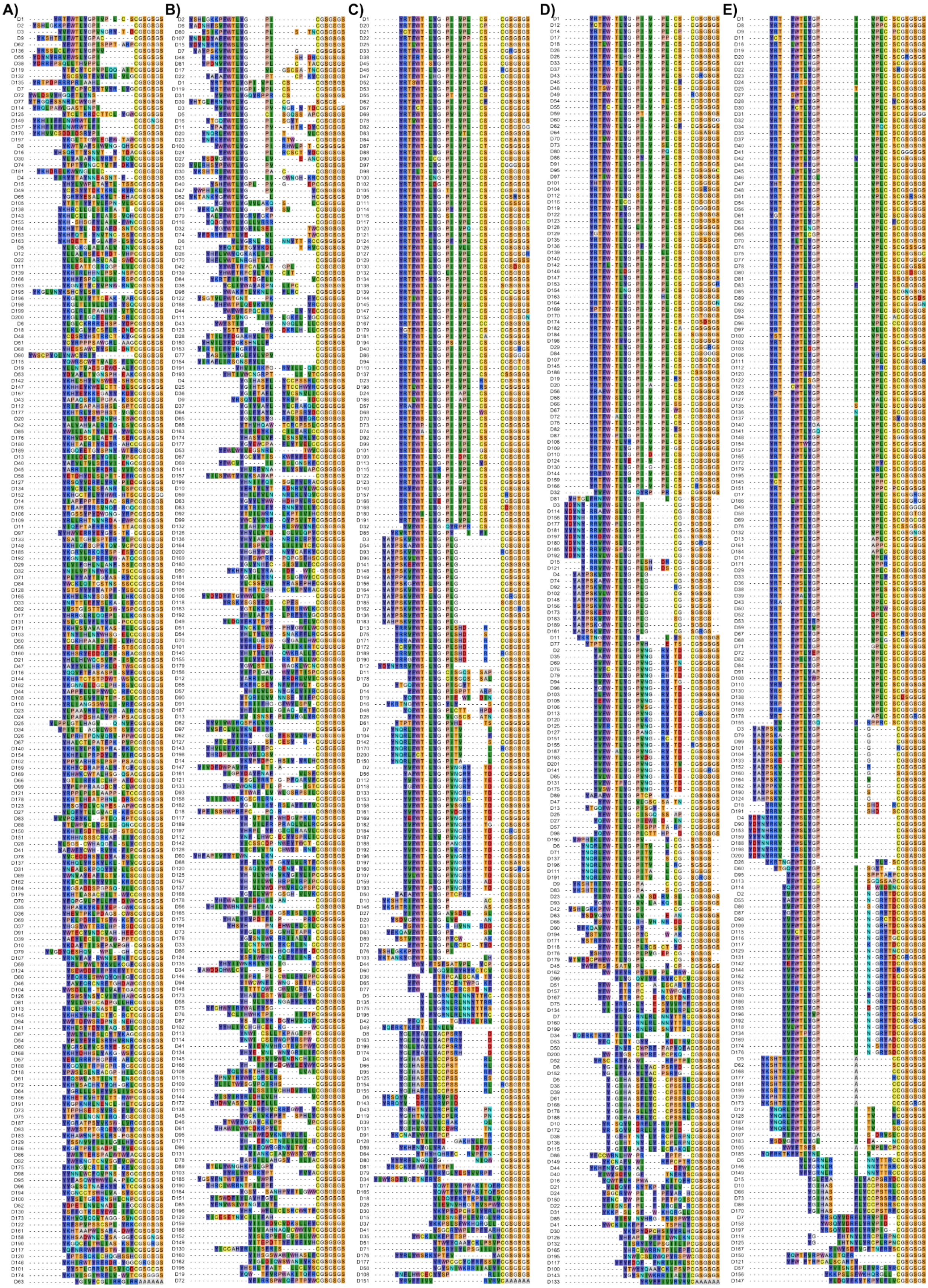

Figure S2. Multiple sequence alignment of the top 200 most abundant sequences in the D-library from round 1 to round 5 (A to E). The preprocessing is done by identification of exact DNA primer sequence matches (T7g10M.F48 and puromycin ligation site), *in silico* translation of each sequence and counting the number of identical peptide sequences. The 'winning' motif is seen already in round 1, and a strong enrichment was observed across later rounds. While D51-54 (54th in round 3) were ranked as the 245th most abundant sequence in round 5, D59-129 (129th in round 3) was not found in the round 5 sequencing data. D28-36 was found as the 36th sequence in round 3 and the 121st sequence in round 5.

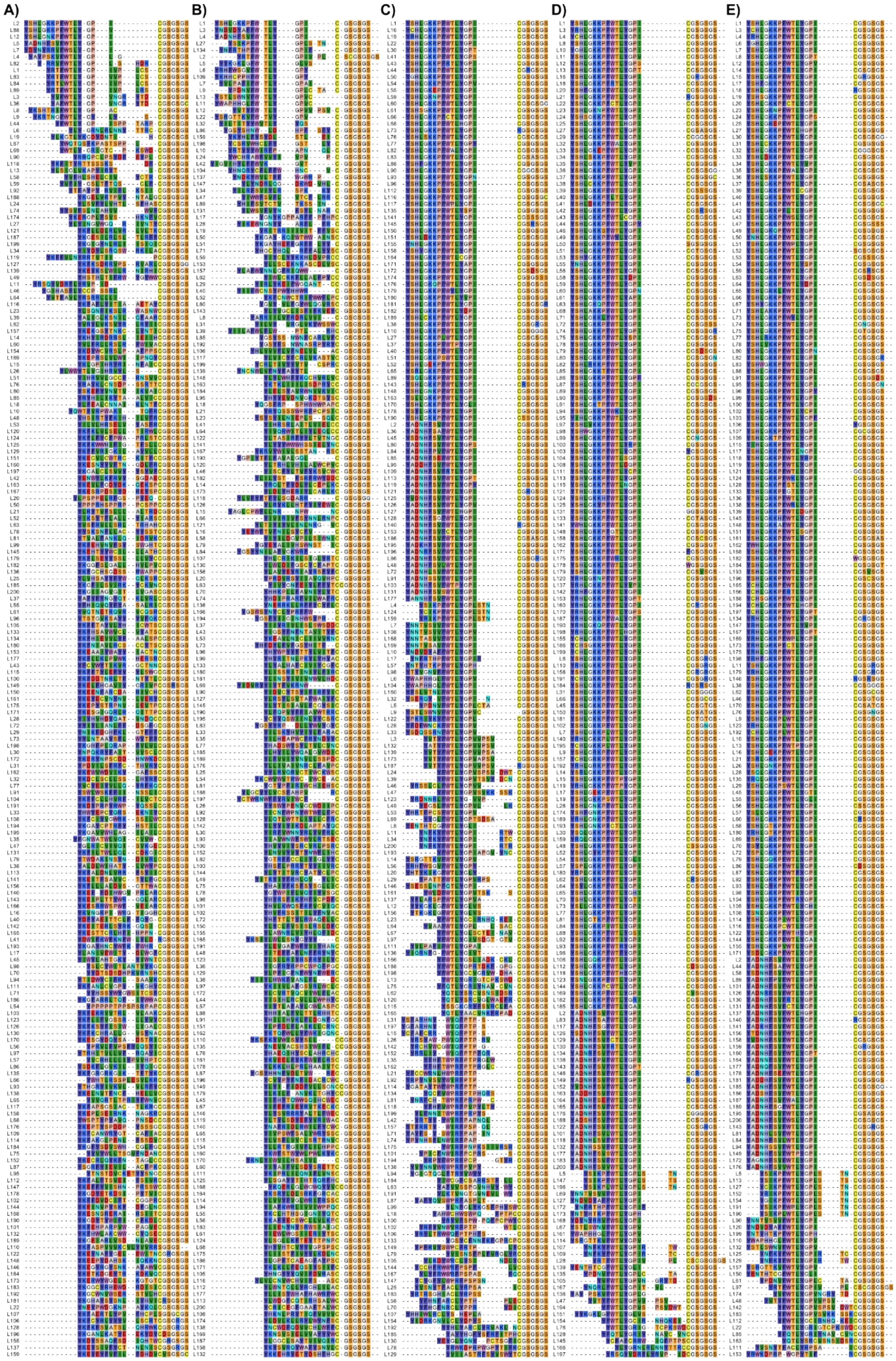

Figure S3. Multiple sequence alignment of the top 200 most abundant sequences in the L-library from round 1 to round 5 (A to E). The preprocessing is done by identification of exact DNA primer sequence matches (T7g10M.F48 and puromycin ligation site), *in silico* translation of each sequence and counting the number of identical peptide sequences. The 'winning' motif is again seen already in round 1, and a strong enrichment was observed across later rounds. While L21-34 (34th in round 3) was not found in round 5, L63-69 (69th in round 3) was ranked at 500th in round 5, and L171-772 (772nd in round 3) was ranked at 3488th in round 5.

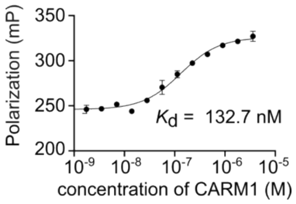

Figure S4. Binding affinity of peptide L1, determined by fluorescence polarization with twofold serial dilution of the CARM1 protein against 50 nM fluorescent L1 probe.

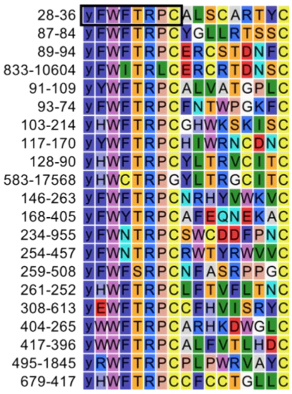

Figure S5. Sequence family with D28-36 as the founding member from our CD-HIT-multiple sequence alignment workflow, illustrating low conservation after the first cysteine. The initiating "y" is the ClAc-D-Tyr residue used for cyclization, with the cyclization moiety omitted from the alignment.

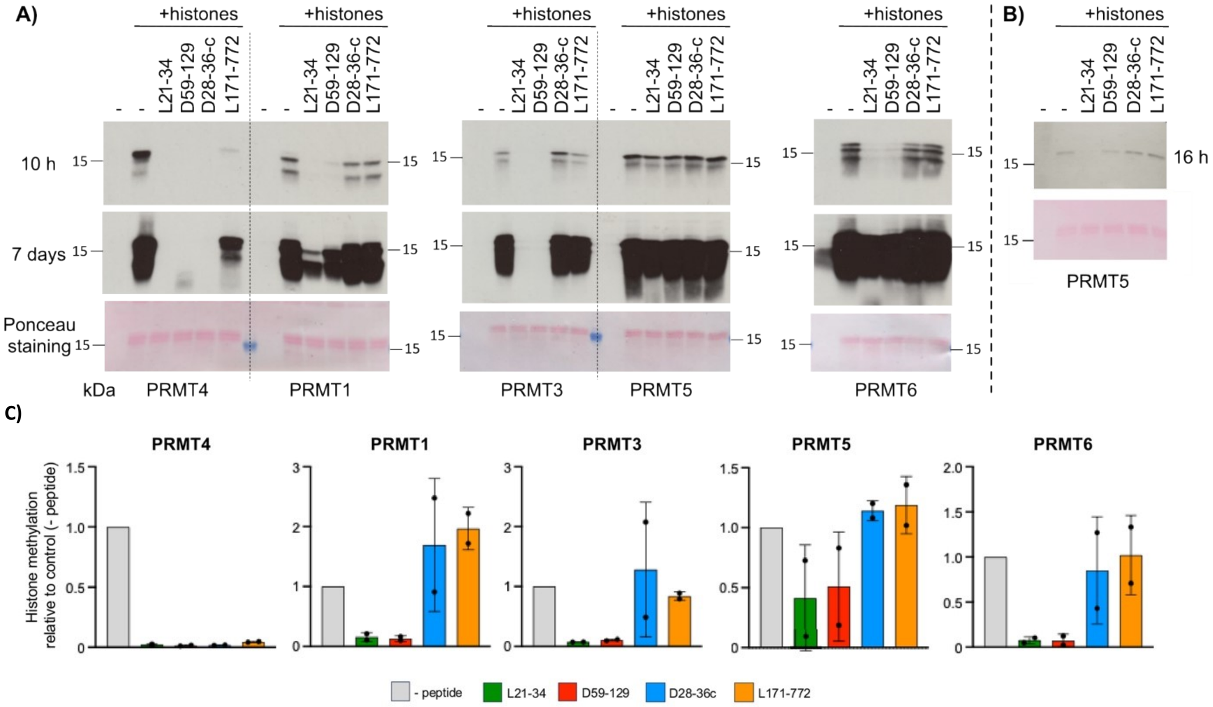

Figure S6. Replicate *in vitro* histone methylation assay with PRMT1, PRMT3, PRMT4, PRMT5, and PRMT6 in the absence (-) or presence of test peptide inhibitors. Methylated proteins are visualized by autoradiography with varied exposure time (specified next to each gel) and histone proteins by Ponceau staining. A) Second replicate (first replicate in Figure 4 of the main text). B) Third replicate for PRMT5 to resolve contradicting replicates 1 and 2. C) The average histone methylation of the two replicates in Figure 4 and S4A was densitometrically quantified using the program Fiji ImageJ and normalized to the corresponding Ponceau staining of the histone proteins. The quantification graphs show the fold changes in PRMT-mediated histone methylation caused by each tested peptide, relative to the control condition (i.e. the methylation reaction - peptide). D28-36-cycle is abbreviated to D28-36-c.

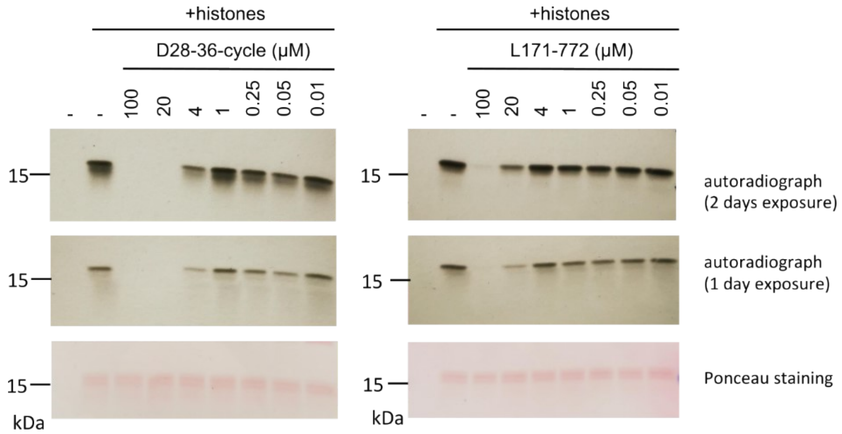

Figure S7. *In vitro* histone protein methylation assay with PRMT4 in the presence of D28-36-cycle and L171-772 at varied concentrations.

Supporting materials and methods

Materials

For peptide synthesis, all standard Fmoc amino acids were purchased from GL biochem (C*hina*). Fmoc-Arg(Me)$_2$(Pbf)-OH and Fmoc-Aha-OH were purchased from Iris Biotech (*Germany*), and Fmoc-Cit-OH was purchased Fluorochem (*United Kingdom*). Fmoc Rink Amide TentaGel resin was purchased from Iris Biotech (*Germany*), DMF was purchased from Merck Group (*Germany*). Pyridine, piperidine, DCM and diethyl ether were purchased from Biosolve Chemie (*France*). Acetic anhydride and DODT were purchased from Sigma-Aldrich (*U.S.A.*). DIC, oxyma pure, HOBt, and HBTU from Manchester Organics (*United Kingdom*). DIPEA was purchased from Carl Roth (*Germany*). TFA was purchased from Apollo Scientific (*United Kingdom*). TIPS was purchased from Fluorochem (*United Kingdom*). Chloroacetic acid was purchased from Thermo Fischer Scientific (*U.S.*A). All analytical HPLC and LC-MS were performed with a 1260 infinity II HPLC in tandem with InfinityLabLC/MSD XT using a C18 column (InfinityLab Poroshell 120 EC-C18, 4.6 × 100 mm, 2.7 µm (Agilent Technologies, *U.S.A*). Suppliers for other reagents and apparatus are specified in the text.

Protein production and immobilization for display

High purity CARM1 protein was produced as an *N*-terminal glutathione S-transferase (GST) fusion with tobacco etch virus (TEV) cleavage site as previously reported[1], using bacmid transfection of Sf9 cells. Purification was by glutathione sepharose column, TEV cleavage, then Superdex S200 gel filtration (GE healthcare). To immobilize the protein for selection, it was exchanged into 20 mM HEPES.KOH pH 7.5, 150 mM NaCl, 0.1 mM DTT using a 30 kDa cut-off spin spin filter and then reacted with 2 equivalents of biotin-(PEG)4-NHS reagent (Thermo Scientific) at 4°C for 2 hours before buffer exchange back into 20 mM Tris-HCl pH 8.0, 100 mM NaCl, 1 mM TCEP. Degree of modification was measured by incubating 200 ng of protein with varying volumes of dynabeads M280 streptavidin (Thermo Scientific) before separation of beads and supernatant, eluting the protein off the beads by incubating in SDS-PAGE loading buffer for 5 min at 95°C, and analyzing on 10% SDS-PAGE with silver staining.

Selection by mRNA display under a reprogrammed genetic code

Selections were carried out as previously reported,[2] using 200 nM biotinylated CARM1 protein during the affinity panning step. Briefly, an RNA library encoding 15 random (NNK) codons flanked by constant regions for T7 RNA polymerase and ribosome binding (5’ end) and encoding a GSGSGS peptide spacer, a stop codon, and a GC rich region for annealing of a puromycin oligonucleotide. Ligation of the puromycin oligonucleotide to the mRNA was achieved by T4 RNA ligase, and the resulting templates translated *in vitro* at 5 µL scale using the PURExpress system (New England Biolabs,

United States of America) by combining □solution A from Δ (aa/tRNA) and solution B from Δ RF123 kits. The resulting peptide-mRNA conjugates were reverse transcribed using □Protoscript II reverse transcriptase and then incubated with first empty beads (3 repeats, increased to 7 in round 4) and subsequently immobilized target protein. These beads were then washed with tris-buffered saline containing 0.01% Tween-20, and the remaining DNA eluted by heat treatment. Samples eluted from the last empty bead portion ('negative'), the target-bound beads ('positive') and from the input library were quantified by qPCR to estimate recovery each round. Eluted cDNA was amplified by PCR, and used for in vitro transcription by T7 RNA polymerase to generate a new mRNA library for the next round. After 5 rounds, all DNA samples were barcoded and submitted for sequencing on the □Illumina MiSeq platform using a 2 X 150 bp V2 reagent kit at the Utrecht UMC sequencing facility (USEQ).

Sequencing data processing workflow

The raw data from next generation sequencing was pre-processed as follows using bash and python scripts in https://github.com/yoshisadades/CARM1_NGS_analysis. 1) Extraction of peptide encoding regions of the sequences (69 bases) that have correct primer (TAATACGACTCACTATAGGGTTAACTTTAAGAAGGAGATATACATATG) sequence and puromycin ligation site (TAGGACGGGGGGCGGAAA) within an error rate of 10% as well as the average quality score below 10. 2) *In silico* translation of the peptide encoding region to the peptide level. 3) Counting identical peptides per round and reporting them based on the abundance ranking at a round of interest.

This abundance summary file of all the relevant sequences was converted to fasta file, then CD-HIT clustering[3–5] was performed with the sequence identity score (argument -c) at 0.65 and the description length limit (argument -d) of 100 in the output FASTA header with output clusters by decreasing length (argument -sc 1). The used command line reads >cd-hit -i [input].fasta -o [output].fasta -c 0.65 -sc 1 -d 100. This classified all of the unique sequences into 877/728 clusters composed of 3 or more sequences, 1,004/772 clusters composed of 2 sequences, and 19,674/7,563 single sequences (with the first number corresponding to the L library and the second number to the D-library, respectively). The analysis was then followed by multiple sequence alignment using only seeds from clusters with 3 or more unique sequences (1,000 seeds). Afterwards, the seed sequence of all clusters with at least 2 members was extracted for the progressive multiple sequence alignment by CLC Sequence Viewer 8 using default settings (gap open cost: 10, gap extension cost: 5, end gap cost: Free, alignment: very accurate).

Automated Solid Phase Peptide Synthesis with microwave aided heating system

Peptide synthesis was performed at 100 µmol scale on CEM HT12 liberty blue peptide synthesizer (CEM corporation, *U.S.A*). The Fmoc Rink Amide AM resin (100 µmol) was swollen with in DMF/DCM (1:1) for 5 minutes, drained then treated with 20% v/v piperidine (10 mL) in DMF for 65 seconds at 90°C, drained and washed three times with DMF (5 mL). The resin was treated with a solution of Fmoc amino acid (0.2 M, 2.5 mL, 5 equivalents), DIC (1 M, 1 mL, 10 equivalents) and Oxyma (1 M, 0.5 mL, 5 equivalents) in DMF (4 mL) at 76°C for 15 seconds before the temperature was increased to 90°C for an additional 110 seconds heating before being drained. The coupling reaction was then repeated for a second time. After the final deprotection of the Fmoc group on the *N*-terminal amino acid, the *N*-terminus was chloroacetylated using 25 µmol of resin with ClAc-NHS in DMF (0.2 M, 1 mL) for 2 × 30 minutes. The resin was washed three times with DMF and then three times with DCM before drying by air flow. The peptide was cleaved from the resin using a cleavage cocktail (containing TFA/water/EDT/TIPS = 92.5:2.5:2.5:2.5) with shaking for 1 hour at 200 rpm. The resin was filtered and peptide in the filtrate was precipitated in pre-chilled ether solution (methyl tert-butyl

ether/petroleum ether = 1:1), vortexed and centrifuged at 6500 rcf for 5 minutes. The pellet was washed twice with the ether solution and dried in air.

Automated Solid Phase Peptide synthesis using induction heating

Peptide synthesis was performed at 25 µmol scale on the PurePrep Chorus synthesizer (Gyros Protein Technologies, *Sweden*). The Fmoc Rink Amide (TentaGel) resin (25 µmol) was swollen with DMF (3 mL) for 3 times 10 minutes at room temperature. Fmoc deprotection was performed with 0.1 M oxyma and 2 M piperidine in DMF (3 mL) with mixing by shaking and nitrogen bubbling for 1.5 minutes at 80°C. The solution was drained and washed with DMF once (3 mL). Subsequent coupling was performed using Fmoc-protected amino acid (0.1 M, 1.25 mL, 5 equivalents) DIC (500 mM, 0.5 mL, 10 equivalents) and oxyma (250 mM, 0.5 mL, 5 equivalents) in DMF (final total volume 3 mL). The reaction solution was mixed by shaking and nitrogen bubbling at 55°C for 15 minutes before the solution was drained. Capping was performed using 2 M acetic anhydride and 2 M pyridine in DMF. (3 mL) with shaking for 5 minutes at room temperature. The resin was then washed three times 3 mL DMF. Chloroacetic acid was coupled as for any amino acid, avoiding final piperidine deprotection. After the final cycle, the resin was washed additionally with DCM (3 mL) and dried with nitrogen flow for 30 minutes.

Peptides were cleaved from the resin using a mixture of TFA/water/TIPS/DODT (90:5:2.5:2.5) with shaking for 3 hours at room temperature. The resin was filtered and washed with TFA (2 mL). The crude peptide was precipitated in pre-chilled diethyl ether (30 mL) on ice and a pellet was obtained by centrifugation (5 minutes at 6500 rpm, 10°C). The pellet was washed twice with diethyl ether (20 mL) and dried in air.

Manual Solid Phase Peptide Synthesis with HBTU/HOBt coupling

The reaction system was composed of a fritted-filter reaction vessel with a three way valve, of which one was connected to nitrogen flow and the other was connected to a 500 mL side arm vacuum flask. Before the synthesis, the vessel was washed with DMF and the Fmoc Rink Amide (TentaGel) resin (25 µmol) was swollen with DMF (3 mL) for 20 minutes at room temperature, The solution was drained and washed with DMF three times 3 mL. Fmoc deprotection was performed with 0.1 M oxyma and 2 M piperidine in DMF (3 mL) for 20 minutes at room temperature. The solution was drained and washed with DMF six times 3 mL. Coupling reaction was performed using Fmoc-protected amino acid (0.1 M, 1 mL, 4 equivalents), HBTU (0.1 M, 1 mL, 4 equivalents), HOBt (0.1 M, 1 mL 4 equivalents) and DIPEA (35 µL, 8 equivalents) in DMF. The reaction was mixed at room temperature by nitrogen bubbling until the coupling was confirmed complete with Kaiser test before being drained and the resin was washed with DMF three times 3 mL. Capping was performed using 2 M acetic anhydride and 2 M pyridine in DMF. (3 mL). The capping solution was drained and the resin was washed with DMF six times (6 × 3 mL). After the final cycle the resin was washed additionally with DCM (3 mL) and dried with nitrogen flow for 30 minutes.

Chloroacetyl cyclization

The dried pellet of crude peptide was dissolved in DMSO (900 µL). To this solution 20 µL of DIPEA was added and gently mixed by shaking. The pH of the solution was confirmed by damp pH paper to be basic (pH ~10), then the solution was left at room temperature for 2 hours and quenched with TFA (20 µL).

HPLC Purification

The crude peptide was loaded on HPLC in DMSO with gradient elution by 10—70% MeCN, 0.1% TFA in water over 50 minutes at 12.5 mL min^{-1}, using 250×21.2 mm packed in 10 µm C18 column

(Phenomenex Gemini). The product fractions were identified by LC-MS and their purity was confirmed to be over 95% with analytical HPLC with the gradient elution by 10—95% MeCN, 0.1% formic acid in water at 0.6 mL min^{-1} over 48 minutes using 2.1×50 mm packing 1.8 μ, C18 column (ZORBAX SB-C18, *Agilent Technologies*, *U.S.A.*), before pure fractions were combined and concentrated by lyophilizer (FreeZone 2.5 Liter -84C Benchtop Freeze Dryer, Labconco*, U.S.A.*) to give a white solid. The dry solids of pure peptides were stored at −20°C until use.

Before use, peptides were dissolved in water and the concentrations were determined using Nanodrop (Thermo Fisher Scientific, *U.S.A.*) with extinction coefficient calculated with the Expasy protparam tool (https://web.expasy.org/protparam/), which uses a direct additive approach.

L1-FAM cyclo[Ac-YSHLGKKPFWTLYGPIC]G-ppX$_{(DBCO\text{-}FAM)}$G-NH_2
Fmoc-G-ppXG-NH_2 (p=PEG based spacer, X=azidohomoalanine; Aha) was synthesized manually with the HBTU/HOBt coupling system using 2-[2-(Fmoc-amino)ethoxy]ethoxyacetic acid (Manchester Organics, *United Kingdom*) and Fmoc-azidohomoalanine (Iris Biotech, *Germany*). The resin was transferred to the automated peptide synthesizer and extended to the full length, followed by cleavage and cyclization to yield cyclo[Ac-YSHLGKKPFWTLYGPIC]G-ppXG-NH_2 (p=PEG, X=azidohomoalanine). The product was purified as described in the general procedure, resulting in a white solid. Equal volumes of 3.6 mM L1-Aha dissolved in PBS buffer (500 mM, pH 7.4) and 5 mM DBCO-FAM (Lumiprobe, *Germany*) in DMSO were mixed and incubated overnight in the dark at room temperature. The product (L1-FAM) was purified by HPLC (column NUCLEODUR C18 ec, 5 µm, 125x10 mm, Macherey Nagel, *Germany*) under gradient elution by 10−70% MeCN, 0.1% TFA in water over 50 minutes at 12.5 mL min^{-1}.

Inhibition assays of original set of peptide by multiple reaction monitoring (MRM) assay
The MRM assay was performed as previously described,[1] detecting methylated PABP1$^{456\text{-}466}$ peptide in LC-MS/MS after reaction with CARM1 and S-adenosylmethionine in the presence of varied concentrations of candidate peptide inhibitor, as compared to an isotopically labeled internal standard.

Fluorescence polarization assay
To each well in a 384-well black plate, 10 µL of reaction solution was loaded containing 1×Tris buffer (20 mM tris, 50 mM NaCl, 1 mM EDTA, 3 mM $MgCl_2$ and 1 mM DTT, pH 8) and 50 nM L1-FAM in water with a series of CARM1 concentrations. The initial concentration of CARM1 was 3.6 µM, followed by subsequent concentrations in a twofold dilution series, totaling 23 concentration steps. A control was prepared by ultrapure water instead of CARM1. The plates were incubated at room temperature for 60 minutes and fluorescence was measured using PHERAstar FS microplate reader (BMG LABTECH, *Germany*) with an excitation wavelength of 485 nm (parallel) and emission wavelength of 520 nm (perpendicular). Data were fit using GraphPad Prism 8.4.3 software to the following equation:

$$Y = Bottom + \frac{X^{Hillslope}(Top - Bottom)}{X^{Hillslope} + EC_{50}{}^{Hillslope}}$$

CARM1 inhibition assay (ELISA)
A 96-well plate coated with PABP1$^{456\text{-}466}$ peptides from the PRMT4 Chemiluminescent assay kit (BPS Bioscience, *U.S.A*) was first rehydrated with 200 µL of 1×TBST buffer (20mM Tris, 150mM NaCl, 0.03% Tween-20, pH 8), followed by incubation at room temperature for 15 min. Subsequently, 50 µL of the pre-mix was added to the wells (containing test peptide in ultrapure water, 1×HMT buffer 5, 1 µM SAM, 0.01% BSA and 200 ng of PRMT4 which was added last to initiate the methylation

reaction) and incubated overnight at room temperature. Test peptide was at 100 µM for the initial scanning of the peptides and sets of 5-times serial dilution ranging from 20–0.032 µM for further characterization of L21-34, D28-36, D59-129 and D28-36-cycle. As a control, ultrapure water was added instead of the test peptide. As a reference, a well that has only 50 µL of 1×HMT buffer was prepared. For detection, chemiluminescence was measured by absorbance at 450 nm and emission at 570 nm using CLARIOStar plus plate reader (BMG LABTECH, *Germany*).

Before testing our peptides, the protocol was validated using competition with synthetic $PABP1^{456\text{-}466}$, resulting in a sigmoid curve with IC_{50} of 3.2 ± 1.96 µM (Figure S8).

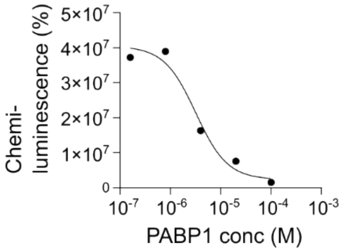

Figure S8. PRMT4 chemiluminescent assay with a control peptide $PABP1^{456\text{-}466}$. The $PABP1^{456\text{-}466}$ IC_{50} was determined as 3.2 ± 1.96 µM.

***In vitro* histone methylation assays with PRMTs.**

Plasmids for generation of Flag-PRMTs-containing baculoviruses

pFASTBAC-Flag-mPRMT4, pFASTBAC-Flag-rPRMT1 and pFASTBAC-Flag-hPRMT5 were previously described.[6–8] The complete ORFs of human PRMT3 and PRMT6 were inserted into the pFASTBAC HT-3xFlag B vector via BamHI/HindIII and BamHI/XhoI sites, respectively. Recombinant Flag-PRMT-containing baculoviral supernatants were generated and used for infection of Sf9 insect cells.

Recombinant protein preparation for specificity testing

For protein preparation of recombinant Flag-tagged PRMTs, baculovirus-infected Sf9 cells were washed twice with PBS prior to 3x freeze and thaw lysis in BC buffer (20 mm HEPES pH 7.9, 250 mm NaCl, 10% glycerin, 0.4 mM EDTA, 1 mM DTT, and 10 µg/µl protease inhibitors). Protein purification was performed using anti-Flag M2 Affinity Gel (Cytiva) as described previously.[6,9] The concentration of recombinant bead-bound PRMT enzymes was determined by SDS/PAGE and Coomassie staining.

In vitro methyltransferase assays with Flag-PRMTs and the test peptides

Recombinant Flag-tagged PRMTs and 10 µg bulk histones from calf thymus (Sigma-Aldrich, *U.S.A.*) were pre-incubated for 30 minutes with the different test peptides (100 µM). The enzymatic reaction was initiated by addition of the methyl donor, [^{14}C-methyl]-S-adenosyl-methionine (^{14}C-methyl-SAM 20 µCi/ml; Perkin Elmer) and performed for 2 hours at 37°C. Reactions were separated by SDS-PAGE, blotted, and analyzed by autoradiography. Radioactive signals were detected using X-ray films (Hyperfilm; Amersham) and intensifying screens (Kodak).

CARM1 inhibition assay with titration of D28-36-cycle and L171-772

Recombinant Flag-tagged PRMT4 and 10 µg bulk histones from calf thymus (Sigma-Aldrich) were pre-incubated for 30 minutes with different concentrations of D28-36-cycle and L171-772 peptides. The enzymatic reaction was carried out as described above.

Substrate direct methylation test (complete methylation)

A reaction mixture with 8.3 µM test peptide, 83 µM AdoMet, 300 nM CARM1 in a buffer at pH 8.0 (containing 20 mM Tris base, 50 mM NaCl, 1 mM EDTA, 3 mM MgCl2, 0.1 mg/mL BSA, and 1mM dithiothreitol (DTT)) was incubated at room temperature for 18 hours and injected to LC-MS. The analysis was performed by extraction of the $[M+2H]^{2+}$ ion masses which correspond to each methylation state of the peptide, followed by integration of each peak.

Substrate direct methylation test (competition)

A reaction mixture with 7.7 µM test peptide, 7.7 µM PABP1$^{456-466}$ substrate, 7.7 µM AdoMet and 277 µM CARM1 in a buffer at pH 8.0 (containing 20 mM Tris base, 50 mM NaCl, 1 mM EDTA, 3 mM MgCl2, 0.1 mg/mL BSA, and 1mM dithiothreitol (DTT)) was incubated at room temperature for 3 hours and injected to LC-MS. The control experiment was performed by replacing the test peptide with ultrapure water. The analysis was performed by extraction of the ion masses which correspond to each methylation state of the peptide, followed by integration of each peak.

NMR structure determination

Unlabeled 1.0 mM L171-772 was dissolved in 130 mM NaCl, 25 mM NaPi pH 6.5, 0.01% NaN_3, 10% D_2O NMR sample buffer. Homonuclear ^{1}H-^{1}H 2D NOESY (200 ms mixing time), 2D TOCSY (80 ms mixing time), 2D COSY-DQF and 2D ^{13}C-^{1}H HSQC were recorded at 293K on a 600 MHz Bruker Avance III HD NMR Spectrometer equipped with a cryo probe (inverse triple resonance with Z-gradient). Typical

acquisition times were 20-50 ms in t_1, 80-280 ms in t_2 and a total acquisition time of 11-40 hours. Spectral processing was performed using Topspin.

^{1}H assignment was carried out using sequential walk[10] based on conventional 2D TOCSY and 2D NOESY spectra, as well as 2D COSY-DQF for regiospecific aromatic assignments and 2D ^{13}C-HSQC for acetyl linker methylene nuclei as well as ^{13}C chemical shifts. Side chain and C-terminal amides were assigned stereospecifically as described by Harsch et al.[11] For assignment and analysis of the spectra, POKY[12] was used. ^{13}C chemical shift referencing was adjusted by 2.66 ppm compared to standard Bruker referencing, as described by Aeschbacher et al.[13] S^2 order parameters and secondary structure propensities were predicted from ^{1}Hα, ^{1}HN, ^{13}Cα and ^{13}Cβ chemical shifts using the TALOS+ web server[14].

Distance restraints were automatically calibrated from NOE peak volumes by CYANA[15] (version 3.98.15) using a reference distance of 4.75 Å. The maximum restraint used was 6 Å. Distances were corrected automatically for the lack of stereospecific assignments in diastereotopic groups. TALOS+ predictions which were marked as 'good' for residues with an order parameter greater than 0.65, as recommended for non-rigid structures, were converted into ϕ and ψ angle torsional restraints of predicted angle ± 2 · standard deviation to allow for limited flexibility and approximate the 95% confidence interval of the prediction. This resulted in 11/9 restraints for the backbone ψ/ϕ dihedral angles, including prolines Pro12 and Pro13. Proline trans conformation was confirmed using Cβ and Cγ chemical shifts as described in literature (P13: 28.162 – 24.449 = 3.713 ppm -> trans; P14: 29.235 – 24.562 = 4.673 ppm -> trans).[16] Additional restraints were applied to restrain the bond geometry of the thioether linker element. Briefly, upper and lower distance limits were set to restrain the S-C bond to 1.8 Å, and additional restraints between the acetyl CH_2 hydrogen and Cys8 sulfur atom (2.4 Å), and between acetyl carbon and the Cys8 CB atom (2.8 Å) were defined to enforce a C-S-C torsion angle of ca. 100°. The peaks and NOE restraints are summarised in Tables S1 through S3.

Structure calculations were performed by restrained torsion angle dynamics using CYANA, starting from 200 randomly generated initial conformations and selecting the 20 lowest energy conformers after 10000 steps. Cyclization was achieved by *i*) modifying the glycine entry in the CYANA library to represent an acetyl group (ACE) with a sulfur atom at the position of the original nitrogen atom in GLY; ii) defining ACE as residue 1 in the sequence; iii) adding a link statement in the sequence file between the acetyl carbon and the Cys8 sulfur atom. The final ensemble of structures showed no violations larger than 0.5 Å or 5°. The linker geometry was inspected visually. Structural statistics are reported in Table S4.

Molecular dynamics

Deriving a sensible bound state proved difficult, as most poses had either the hydrophobic face of the macrocycle exposed to solution or the tail outside of the substrate-binding cleft. Docking of the NMR structure or *ab initio* predictions with Haddock[56], Gnina[57], or Alphafold 2[58] (with a linearized peptide), proved to be inefficient starting points for molecular dynamics simulations. A novel composite-based approach was therefore used, in which docked poses for each of the macrocycle and the tail were combined before molecular dynamics of the entire peptide (with the final structure having acceptable dihedral angles at this ring-tail junction).

The X-ray crystallographic structure of CARM1 (PDB ID: 5DXA, resolution 2.07 Å)[17] was used as the starting point for molecular dynamics simulations. The structure was initially processed using pdb4amber (AmberTool24),[18,19] retaining only chain A and removing all hydrogen atoms. The processed structure was subsequently protonated using ChimeraX (v.10.7)[20–22] at pH 7.0. Histidine residues were renamed to reflect their protonation states (HID for δ-protonated, HIE for ε-

protonated). Finally, the substrate/ligand peptide and CARM1 enzyme were separated into individual files for downstream parameterization and simulation setup.

Initial CARM1–L171-772 complexes were generated using molecular docking with Gnina (v.1.0),[23] employing the *Vinardo* scoring function[24] and the default convolutional neural network (CNN) model. The docking search space was defined using a box automatically generated around the crystallographic substrate. Docking was performed with an exhaustiveness of 128, and the top 40 binding poses were selected based on their CNN scores. The resulting complex structures were imported into tleap (AmberTools24) to generate coordinate and topology files for implicit-solvent molecular dynamics simulations using the Amber19 force field.

Each complex structure was initially energy-minimized in three successive rounds using Amber24: (i) 100 steps of steepest descent, (ii) 100 steps of conjugate gradient, and (iii) 20 steps of final minimization. Minimizations were performed in a standard Generalized Born (GB) implicit solvent environment with gb=5 and gbsa=1. The minimized structures were then subjected to molecular dynamics simulations using the GPU-accelerated pmemd-cuda program (gb=5, gbsa=3) for 10 ns. Using hydrogen mass repartitioning,[25] all simulations were run with a 4 fs time step and the temperature maintained at 300 K using Langevin dynamics (ntt=3).

Trajectories were analyzed using MMGBSA to decompose residue-level contributions to binding, and MMPBSA[26] was used to estimate the binding affinity of L171-772. Visual inspection of the final frames of each trajectory (ChimeraX) indicated that the initial binding poses often exhibited partial dissociation of either the cyclic or linear domains of the peptide.

To address this, strongly binding linear and cyclic domains that were geometrically compatible were manually combined. Geometric compatibility was assessed based on the relative orientation of the linear and cyclic domains. In total, three linear and two cyclic domains that fit these criteria were selected and combined to generate six new model structures, which were subjected to the same minimization and molecular dynamics protocol described above.

The resulting trajectories displayed improved stability, and these final models were subsequently analyzed using MMGBSA (for residue decomposition) and MMPBSA (for overall binding energies), with the more accurate MMPBSA values reported in the main text.

An archive of all input files and structures is further provided in the electronic supporting information.

Table S1. NMR peak table for L171-772 (before 2.66 ppm ^{13}C correction)

Residue	Atom	Chemical shift (ppm)
Ace1	CA	34.937
Ace1	HA2	3.13
Ace1	HA3	3.095
Y2	CA	55.45
Y2	CB	36.001
Y2	CD1	130.272
Y2	CE1	115.515
Y2	H	8.263
Y2	HA	4.423
Y2	HB2	2.717
Y2	HB3	2.78

Y2	QD	6.848
Y2	QE	6.599
W3	CA	55.147
W3	CB	26.398
W3	CD1	124.379
W3	CE3	118.161
W3	CH2	121.945
W3	CZ2	111.957
W3	CZ3	119.376
W3	H	8.017
W3	HA	4.426
W3	HB2	3.071
W3	HB3	3.098
W3	HD1	7.068
W3	HE1	10.086
W3	HE3	7.35
W3	HH2	7.105
W3	HZ2	7.357
W3	HZ3	7.001
K4	CA	54.389
K4	CB	29.43
K4	CD	26.318
K4	CE	39.337
K4	CG	21.744
K4	H	7.314
K4	HA	3.773
K4	HB2	1.264
K4	HB3	1.469
K4	HD2	1.35
K4	HD3	1.377
K4	HE2	2.705
K4	HE3	2.723
K4	HG2	0.776
K4	HG3	0.805
D5	CA	51.922
D5	CB	38.002
D5	H	7.827
D5	HA	4.323
D5	HB2	2.483
D5	HB3	2.585
F6	CA	55.956
F6	CB	36.24
F6	CD1	129.094
F6	CE1	128.756
F6	CZ	127.113
F6	H	7.88
F6	HA	4.327
F6	HB2	3.021
F6	HB3	3.057
F6	HD	7.114
F6	HE	7.229
F6	HZ	7.172
I7	CA	59.087
I7	CB	35.504

I7	CD1	9.93
I7	CG1	25.037
I7	CG2	14.789
I7	H	7.913
I7	HA	3.935
I7	HB	1.738
I7	HG12	1.011
I7	HG13	1.318
I7	QD1	0.682
I7	QG2	0.748
R8	CA	53.511
R8	CB	28.03
R8	CD	40.622
R8	CG	24.685
R8	H	7.961
R8	HA	4.114
R8	HB2	1.562
R8	HB3	1.689
R8	HG2	1.436
R8	HG3	1.467
R8	QD	2.983
C9	CA	53.365
C9	CB	33.078
C9	H	8.07
C9	HA	4.37
C9	HB2	2.735
C9	HB3	2.758
I10	CA	58.339
I10	CB	36.039
I10	CD1	9.868
I10	CG1	24.325
I10	CG2	14.671
I10	H	7.931
I10	HA	4.03
I10	HB	1.685
I10	HG12	0.984
I10	HG13	1.206
I10	QD1	0.678
I10	QG2	0.692
Y11	CA	55.321
Y11	CB	36.114
Y11	CD1	130.361
Y11	CE1	115.282
Y11	H	8.124
Y11	HA	4.382
Y11	HB2	2.759
Y11	HB3	2.829
Y11	QD	6.924
Y11	QE	6.652
R12	CA	50.164
R12	CB	28.108
R12	CD	40.525
R12	CG	23.962
R12	H	7.782

R12	HA	4.379
R12	HB2	1.426
R12	HB3	1.561
R12	HG2	1.393
R12	HG3	1.409
R12	QD	2.981
P13	CA	58.469
P13	CB	28.162
P13	CD	47.796
P13	CG	24.449
P13	HA	4.375
P13	HB2	1.768
P13	HB3	2.206
P13	HD2	3.39
P13	HD3	3.41
P13	HG2	1.852
P13	HG3	1.87
P14	CA	60.125
P14	CB	29.235
P14	CD	47.732
P14	CG	24.562
P14	HA	4.27
P14	HB2	1.742
P14	HB3	2.126
P14	HD2	3.53
P14	HD3	3.659
P14	HG2	1.893
P14	HG3	1.908
I15	CA	58.336
I15	CB	35.939
I15	CD1	10.19
I15	CG1	24.459
I15	CG2	14.59
I15	H	8.059
I15	HA	3.988
I15	HB	1.677
I15	HG12	1.034
I15	HG13	1.348
I15	QD1	0.709
I15	QG2	0.735
I16	CA	58.067
I16	CB	36.146
I16	CD1	10.052
I16	CG1	24.59
I16	CG2	14.751
I16	H	8.14
I16	HA	4.044
I16	HB	1.707
I16	HG12	1.04
I16	HG13	1.309
I16	QD1	0.72
I16	QG2	0.742
D17	CA	51.351
D17	CB	38.579

D17	H	8.295
D17	HA	4.468
D17	HB2	2.475
D17	HB3	2.58
A18	CA	50.165
A18	CB	16.222
A18	H	8.329
A18	HA	4.153
A18	QB	1.281
G19	CA	42.312
G19	H	8.354
G19	QA	3.76

Table S2. Restraints for L171-772 structural refinement.

Residue 1	atom	shift (ppm)	Residue 2	atom	shift (ppm)	Intensity
K4	HB2	1.262	Y2	QE	6.597	1.26E+05
K4	HB3	1.473	Y2	QE	6.6	8.83E+04
K4	HB2	1.263	D5	H	7.827	1.52E+05
K4	HB3	1.469	D5	H	7.827	2.13E+05
R12	HB2	1.415	R12	H	7.783	4.30E+05
I10	HG13	1.205	I10	H	7.932	1.93E+05
I7	HG13	1.318	I7	H	7.914	2.99E+05
R12	HB3	1.56	R12	H	7.783	2.46E+05
I7	HG12	1.01	I7	H	7.917	3.51E+05
I10	HG12	0.986	I10	H	7.931	3.12E+05
I15	HG12	1.033	I15	H	8.06	2.66E+05
I16	HG12	1.042	I16	H	8.142	1.94E+05
I16	HG13	1.308	I16	H	8.141	1.53E+05
I15	HG13	1.356	I15	H	8.061	2.83E+05
R8	HB2	1.562	R8	H	7.962	5.69E+05
R8	HG2	1.444	R8	H	7.962	2.10E+05
I7	QG2	0.749	R8	H	7.961	3.86E+05
I7	QG2	0.749	I7	H	7.914	4.19E+05
I10	QG2	0.692	I10	H	7.931	3.58E+05
I15	QG2	0.739	I15	H	8.061	3.67E+05
I10	QG2	0.69	Y11	H	8.124	4.52E+05
I15	QG2	0.739	I16	H	8.142	5.01E+05
I16	QG2	0.741	D17	H	8.295	2.21E+05
I16	HB	1.709	D17	H	8.294	1.15E+05
K4	HB2	1.259	K4	H	7.311	1.06E+05
K4	HB3	1.474	K4	H	7.314	7.89E+04
I16	HB	1.703	I16	H	8.139	7.26E+05
R8	HG2	1.457	C9	H	8.073	7.32E+04
R8	HB2	1.56	C9	H	8.07	8.65E+04
I10	HG13	1.206	Y11	H	8.124	8.63E+04
I7	HB	1.741	I7	H	7.914	8.97E+05
I15	HB	1.676	I15	H	8.061	1.01E+06
P14	HB2	1.735	I15	H	8.061	2.96E+05
I7	HB	1.738	R8	H	7.96	3.56E+05
I10	HB	1.685	I10	H	7.935	5.53E+05
I10	QG2	0.693	Y11	QE	6.654	1.23E+05

P13	HB3	2.205	Y11	QE	6.65	2.02E+05
D5	HB2	2.483	D5	H	7.827	2.85E+05
D5	HB3	2.584	D5	H	7.829	2.21E+05
D5	HB2	2.484	F6	H	7.88	2.86E+05
D5	HB3	2.585	F6	H	7.88	2.33E+05
Y2	HB3	2.777	Y2	H	8.264	2.73E+05
Y11	HB3	2.829	Y11	H	8.124	6.98E+05
Y11	HB2	2.759	Y11	H	8.124	7.64E+05
D17	HB2	2.475	D17	H	8.295	1.49E+05
D17	HB3	2.583	D17	H	8.295	1.55E+05
Y2	HB3	2.78	W3	H	8.017	2.00E+05
Y11	HB2	2.76	R12	H	7.781	1.56E+05
Y11	HB3	2.83	R12	H	7.783	1.50E+05
Y2	HB2	2.717	Y2	QD	6.847	1.15E+06
Y11	HB2	2.76	Y11	QD	6.923	1.87E+06
Y11	HB3	2.828	Y11	QD	6.923	1.50E+06
Y2	HB3	2.781	Y2	QD	6.849	1.18E+06
Y2	HB3	2.78	Y2	QE	6.603	2.42E+05
Y2	HB2	2.717	Y2	QE	6.603	2.63E+05
Y11	HB3	2.829	Y11	QE	6.656	2.61E+05
Y11	HB2	2.76	Y11	QE	6.656	2.97E+05
G19	QA	3.762	G19	H	8.356	6.73E+05
I16	HA	4.045	D17	H	8.296	1.47E+06
I10	HA	4.034	Y11	H	8.127	2.72E+06
I15	HA	3.993	I16	H	8.142	2.73E+06
K4	HA	3.774	D5	H	7.826	7.04E+05
I7	HA	3.936	R8	H	7.962	1.61E+06
I7	HA	3.936	I7	H	7.912	6.58E+05
I15	HA	3.99	I15	H	8.055	9.87E+05
I10	HA	4.033	I10	H	7.927	4.42E+05
R8	HA	4.117	R8	H	7.958	5.47E+05
R8	HA	4.117	C9	H	8.069	7.45E+05
P14	HA	4.272	I15	H	8.061	3.16E+06
A18	HA	4.156	A18	H	8.326	3.01E+05
D17	HA	4.471	A18	H	8.33	4.20E+05
D17	HA	4.469	D17	H	8.292	3.98E+05
Y2	HA	4.423	Y2	H	8.262	2.20E+05
Y11	HA	4.384	Y11	H	8.122	5.75E+05
W3	HA	4.425	W3	H	8.018	1.22E+06
C9	HA	4.371	C9	H	8.067	3.96E+05
C9	HA	4.372	I10	H	7.933	1.26E+06
F6	HA	4.328	I7	H	7.91	1.01E+06
F6	HA	4.327	F6	H	7.881	2.37E+06
D5	HA	4.326	D5	H	7.828	7.48E+05
W3	HA	4.428	W3	HE3	7.345	6.45E+05
W3	HA	4.428	K4	H	7.316	3.49E+05
K4	HA	3.774	K4	H	7.316	2.30E+05
F6	HA	4.328	F6	HD	7.114	7.96E+05
W3	HA	4.428	W3	HD1	7.069	6.80E+05
Y11	HA	4.383	Y11	QD	6.922	1.39E+06
Y2	HA	4.424	Y2	QD	6.847	9.04E+05
Y11	HA	4.381	Y11	QE	6.654	4.88E+05
Y2	HA	4.425	Y2	QE	6.602	2.31E+05
Y2	QD	6.849	Y2	H	8.262	1.20E+05

Y11	QD	6.928	Y11	H	8.124	2.38E+05
Y2	QD	6.849	W3	H	8.018	1.10E+05
Y11	QD	6.928	R12	H	7.781	2.09E+05
W3	HD1	7.068	W3	H	8.018	2.00E+05
F6	HD	7.117	F6	H	7.88	2.72E+05
W3	HE3	7.351	W3	H	8.018	1.31E+05
R12	H	7.785	Y11	H	8.123	1.22E+05
Y11	QE	6.651	Y11	QD	6.918	1.46E+07
Y2	QE	6.6	Y2	QD	6.843	1.76E+07
W3	HZ2	7.359	W3	HE1	10.086	9.58E+05
W3	HD1	7.069	W3	HE1	10.086	2.95E+06
I7	HG12	1.013	I7	HA	3.935	3.07E+05
K4	HB3	1.472	K4	HA	3.77	7.65E+05
I7	QG2	0.747	I7	HA	3.934	9.59E+05
I16	QG2	0.74	I16	HA	4.042	4.54E+05
I15	QG2	0.736	I15	HA	3.987	6.58E+05
I10	QG2	0.69	I10	HA	4.031	1.18E+06
K4	HB2	1.267	K4	HA	3.771	2.36E+05
I7	HG13	1.319	I7	HA	3.935	2.22E+05
R8	HB2	1.563	R8	QD	2.981	6.05E+05
R8	HB3	1.689	R8	QD	2.984	4.66E+05
R12	HG3	1.408	R12	QD	2.982	1.83E+06
P14	HB2	1.756	P14	HD2	3.532	3.84E+05
P14	HB2	1.75	P14	HD3	3.657	2.52E+05
R8	HB3	1.69	R8	HA	4.109	3.58E+05
I7	HB	1.744	I7	HA	3.941	4.81E+05
I15	HB	1.675	I15	HA	3.973	5.80E+05
I10	HB	1.688	I10	HA	4.021	7.34E+05
P13	HB3	2.206	P14	HD2	3.532	2.70E+05
P14	HB2	1.743	P14	HB3	2.133	1.73E+06
P13	HB2	1.77	P13	HB3	2.213	2.49E+06
K4	HB2	1.264	K4	HB3	1.461	3.91E+06
I10	HG12	0.986	I10	HG13	1.199	4.04E+06
I16	HG12	1.039	I16	HG13	1.307	7.82E+06
R8	HB2	1.564	R8	HB3	1.683	5.67E+06
I10	QG2	0.698	I10	HB	1.69	1.45E+06
I7	HG13	1.315	I7	HB	1.736	6.10E+05
I10	HG13	1.209	I10	HB	1.686	5.30E+05
P14	HB3	2.126	P14	HD3	3.659	1.92E+05
P13	HB3	2.205	P14	HD3	3.657	1.71E+05
P14	HB3	2.123	P14	HD2	3.534	1.52E+05
P14	HD2	3.527	P13	HA	4.373	1.40E+05
P14	HD3	3.66	P13	HA	4.373	1.81E+05
I10	QG2	0.691	Y11	QD	6.925	1.71E+05
I15	HG12	1.035	I15	HA	3.988	1.63E+05
I10	HG12	0.986	I10	HA	4.031	2.07E+05
I10	HG13	1.207	I10	HA	4.03	1.47E+05
Y11	HB3	2.83	R12	HA	4.378	1.99E+05
R12	HA	4.38	R12	H	7.782	2.91E+06
I15	HG12	1.035	I15	HB	1.684	6.70E+05
I7	QG2	0.746	I7	HB	1.726	1.20E+06
D5	HB3	2.589	D5	HA	4.324	3.42E+05
I16	QG2	0.74	D17	HB2	2.482	1.89E+05
I10	QG2	0.696	Y11	HB2	2.754	1.85E+05

I15	QG2	0.722	I15	HG13	1.333	3.91E+06
I10	QG2	0.691	I10	HG13	1.214	2.41E+06
P14	HB3	2.125	I15	H	8.06	1.87E+05
K4	HG3	0.805	K4	HA	3.774	2.81E+05
K4	HG2	0.776	K4	HA	3.773	2.91E+05
Ace1	HA3	3.095	Y2	H	8.263	3.51E+05
Ace1	HA2	3.135	Y2	H	8.263	3.71E+05
W3	HB2	3.071	W3	H	8.017	4.37E+05
W3	HB3	3.098	W3	H	8.017	4.62E+05
W3	HB3	3.098	W3	HE3	7.348	6.68E+05
W3	HB2	3.071	W3	HE3	7.348	6.51E+05
W3	HB3	3.098	W3	HD1	7.068	1.07E+06
W3	HB2	3.07	W3	HD1	7.068	9.78E+05
F6	HB3	3.057	I7	H	7.909	2.88E+05
F6	HB2	3.021	I7	H	7.91	2.65E+05
F6	HB2	3.021	F6	H	7.881	7.82E+05
F6	HB3	3.057	F6	H	7.881	7.17E+05
F6	HB3	3.057	F6	HE	7.223	2.69E+05
F6	HB2	3.021	F6	HE	7.223	2.07E+05
F6	HB3	3.057	F6	HD	7.113	1.07E+06
F6	HB2	3.021	F6	HD	7.113	9.02E+05
C9	HB3	2.76	I10	H	7.932	2.20E+05
C9	HB2	2.737	I10	H	7.932	2.46E+05
C9	HB3	2.761	C9	H	8.069	3.42E+05
C9	HB2	2.737	C9	H	8.069	4.82E+05
C9	HB3	2.761	C9	HA	4.371	3.10E+05
C9	HB2	2.738	C9	HA	4.372	3.37E+05
Y2	HB2	2.716	Y2	H	8.263	3.34E+05
Y2	HB2	2.717	W3	H	8.017	2.02E+05
Y2	HB2	2.718	W3	HD1	7.069	1.28E+05
Y2	HB3	2.777	W3	HD1	7.069	1.26E+05
K4	HE3	2.722	W3	HE3	7.352	1.56E+05
K4	HE2	2.706	W3	HE3	7.353	1.31E+05
R8	HG3	1.464	R8	QD	2.982	1.03E+06
P13	HG3	1.871	Y11	QE	6.649	1.16E+05
P13	HG2	1.849	Y11	QE	6.649	1.54E+05
K4	HD2	1.351	Y2	QE	6.596	7.43E+04
K4	HD3	1.377	Y2	QE	6.595	9.08E+04
P13	HB3	2.204	P13	HD3	3.413	3.10E+05
P13	HB3	2.204	P13	HD2	3.39	2.06E+05
P13	HB2	1.769	P13	HD2	3.39	3.74E+05
P14	HG2	1.891	P14	HD3	3.658	1.02E+06
P14	HG3	1.911	P14	HD3	3.658	1.60E+06
P14	HG2	1.891	P14	HD2	3.531	1.28E+06
P14	HG3	1.911	P14	HD2	3.531	1.51E+06
A18	QB	1.283	A18	HA	4.146	9.27E+05
P13	HG2	1.849	P13	HD3	3.41	1.63E+06
P13	HG2	1.849	P13	HD2	3.39	1.46E+06
P13	HB2	1.769	P13	HD3	3.409	5.11E+05
P13	HG3	1.869	P13	HD2	3.39	1.32E+06
P13	HG3	1.869	P13	HD3	3.41	2.36E+06
R12	HG3	1.407	P13	HD3	3.409	2.49E+05
R12	HG3	1.407	P13	HD2	3.389	1.70E+05
R8	HG3	1.464	R8	HA	4.112	1.33E+05

R8	HG2	1.42	R8	HA	4.11	1.70E+05
R8	HG2	1.422	R8	QD	2.982	1.67E+06
R12	HB3	1.563	P13	HD3	3.409	3.55E+05
R12	HB3	1.564	P13	HD2	3.389	2.42E+05
P13	HD2	3.391	Y11	QD	6.923	1.23E+05
P13	HD3	3.41	Y11	QD	6.921	1.93E+05
P13	HD2	3.391	Y11	QE	6.655	1.22E+05
P13	HD3	3.41	Y11	QE	6.655	1.49E+05
P14	HG3	1.911	I15	H	8.061	1.07E+05
P14	HG2	1.894	I15	H	8.061	1.22E+05
P13	HD2	3.391	R12	H	7.782	8.41E+04
P13	HD3	3.41	R12	H	7.782	1.08E+05
P13	HD2	3.391	P13	HA	4.373	2.21E+05
P13	HD3	3.41	P13	HA	4.373	2.76E+05
C9	HB2	2.733	Ace1	HA3	3.095	5.16E+05
C9	HB3	2.756	Ace1	HA3	3.095	5.49E+05
C9	HB2	2.733	Ace1	HA2	3.126	6.11E+05
C9	HB3	2.755	Ace1	HA2	3.124	6.49E+05

Table S3. Derived angle restrains from TALOS+

Residue	Type	Angle	Uncertainty
W3	PHI	-120.794	-49.97
W3	PSI	108.529	166.763
K4	PHI	-120.638	-32.39
K4	PSI	105.717	165.279
I7	PHI	-129.669	-59.503
I7	PSI	107.52	152.11
C9	PHI	-149.106	-112.766
C9	PSI	138.631	176.617
I10	PHI	-124.688	-61.444
I10	PSI	104.927	147.013
R12	PHI	-117.73	-62.148
R12	PSI	125.365	170.313
P14	PSI	131.778	169.366
P14	PSI	132.904	165.936
I15	PHI	-146.789	-75.457
I15	PSI	93.845	153.149
I16	PHI	-143.328	-83.986
I16	PSI	113.666	148.948
D17	PHI	-125.969	-42.015
D17	PSI	96.68	157.638

Table S4. Structural statistics for L171-772

A. Restraint information	
Total number of distance restraints	92
intra-residual/sequential/medium/long	34/42/12/4
Total number of linker distance restraints (upper/lower)	4/4
Total number of backbone dihedral angle restraints ψ/ϕ	11/9
B. Average deviation from experimental restraints	
RMS experimental distance restraints (Å)	0.0267 ± 0.0012
Average number of distance violations > 0.5 Å	0

RMS experimental dihedral angle restraints (°)	0.64 ± 0.34
Average number of dihedral angle violations > 5°	0
C. Coordinate RMS deviation (Å)	
Average overall RMSD to mean structure[a]	
Ring heavy backbone atoms	0.67 ± 0.23
Ring all heavy atoms	1.95 ± 0.49
Tail heavy backbone atoms	0.96 ± 0.29
Tail all heavy atoms	1.51 ± 0.26
Global backbone atoms	2.58 ± 0.98
Global all heavy atoms	3.46 ± 1.00
D. Ramachandran plot quality parameters (%)	
Residues in most favoured regions	68.2
Residues in allowed regions	14.6
Residues in additionally allowed regions	17.6
Residues in disallowed regions	0.0
E. Abnormalities found in structural checks	
Abnormally short interatomic distances	1

[a] statistics are given for residues 1-18. Ring region is residues 1-8. Tail region is residues 9-18

Peptide characterization

L1. Cyclo[Ac-YSHLGKKPFWTLYGPIC]G-NH_2

LC-MS: (+ESI) m/z Calculated mass for $[C_{102}H_{144}N_{24}O_{23}S + H]^+$: 2106.0630, found: 1053.5357 $[M+2H]^{2+}$, deconvoluted: 2106.07.

Analytical HPLC: Retention time (Rt) = 9.5 min (10-70 vol.% MeCN in H_2O with 0.1 vol.% FA over 20 min, λ = 214 nm).

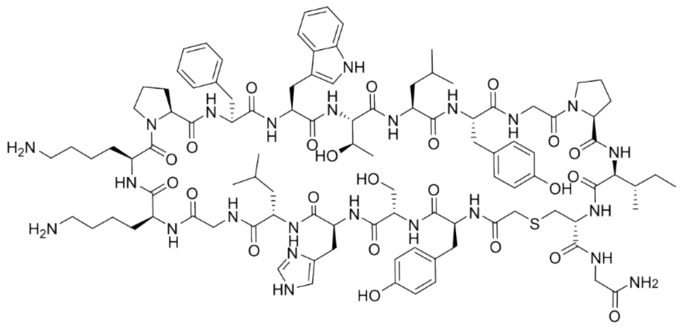

Cyclo[Ac-YSHLGKKPFWTLYGPIC]G-NH_2

Exact Mass: 2105.0557

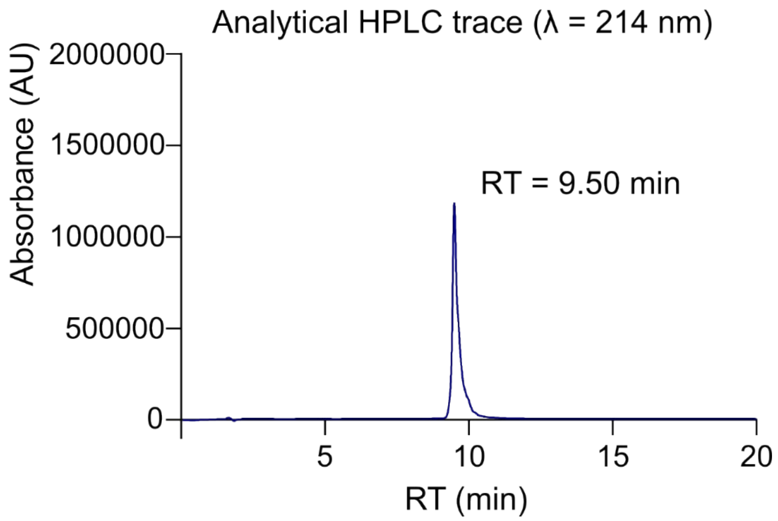

L2. Cyclo[Ac-YADNHFSVFWTLYGPIC]G-NH_2
LC-MS: (+ESI) m/z Calculated mass for $[C_{103}H_{135}N_{23}O_{26}S +H]^{+}$: 2142.9703, found: 1071.9920 $[M+2H]^{2+}$, deconvoluted: 2142.98.
Analytical HPLC: Retention time (Rt) = 11.09 min (10-70 vol.% MeCN in H_2O with 0.1 vol.% FA over 20 min, λ = 214 nm).

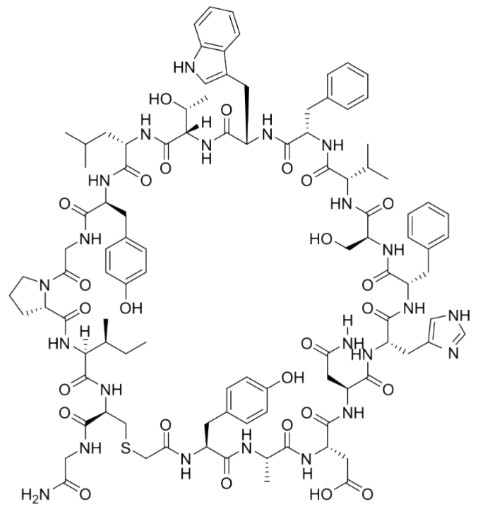

Cyclo[Ac-YADNHFSVFWTLYGPIC]G-NH_2

Exact Mass: 2127.9513

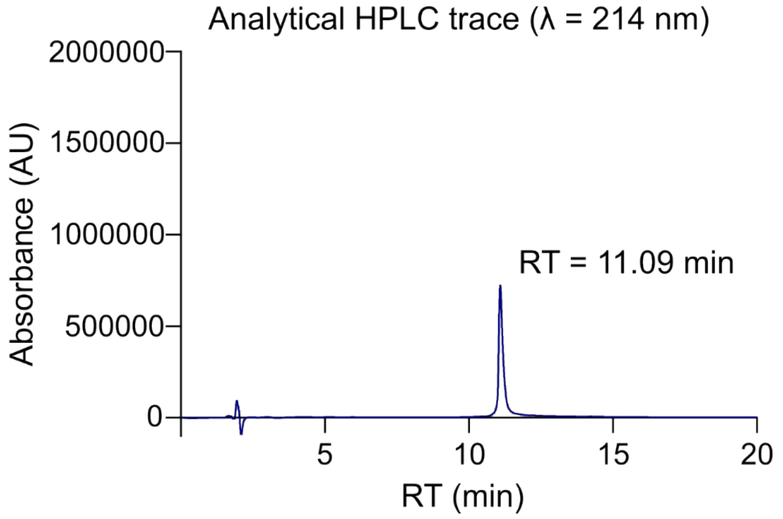

L4. Cyclo[Ac-YRHLGKKPFWTLYGPIC]G-NH_2

RLC-MS: (+ESI) m/z Calculated mass for $[C_{105}H_{151}N_{27}O_{22}S + H]^+$: 2175.1320, found: $[M+2H]^{2+}$ 1088.0709, deconvoluted: 2175.14.

Analytical HPLC: Retention time (Rt) = 9.21 min (10-70 vol.% MeCN in H_2O with 0.1 vol.% FA over 20 min, λ = 214 nm).

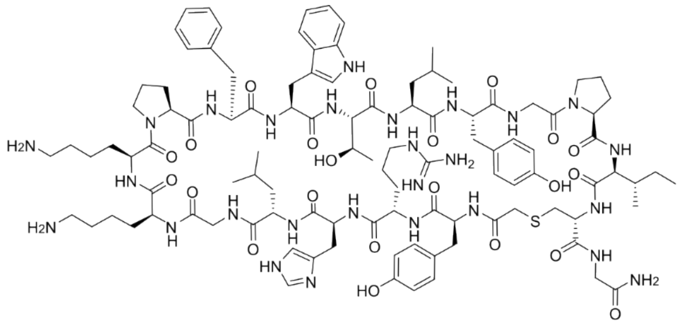

Cyclo[Ac-YRHLGKKPFWTLYGPIC]G-NH_2

Exact Mass: 2174.1248

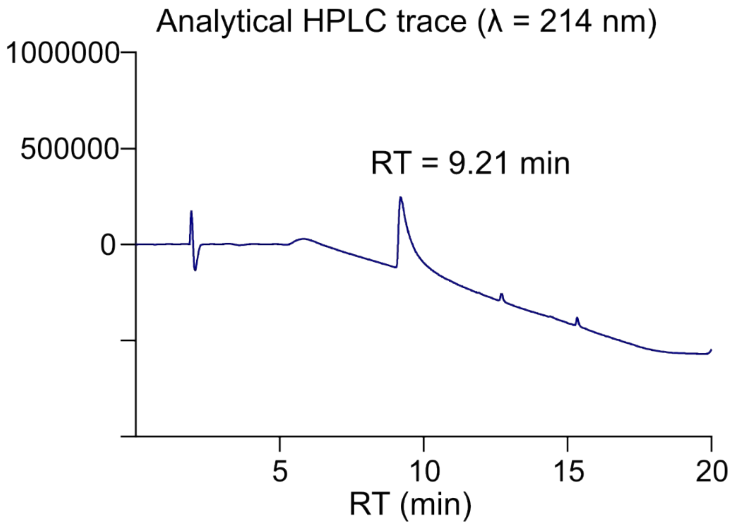

L6. Cyclo[Ac-YGHLGKKPFWTLYGPIC]G-NH_2

LC-MS: (+ESI) m/z Calculated mass for [$C_{101}H_{142}N_{24}O_{22S}$ +H]$^+$: 2076.0524, found: [M+2H]$^{2+}$ 1038.5327, deconvoluted: 2076.07.

Analytical HPLC: Retention time (Rt) = min (10-70 vol.% MeCN in H_2O with 0.1 vol.% FA over 20 min, λ = 214 nm).

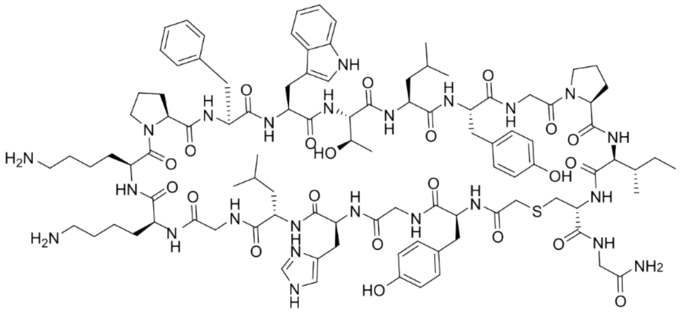

Cyclo[Ac-YGHLGKKPFWTLYGPIC]G-NH_2

Exact Mass: 2075.0451

Analytical HPLC trace (λ = 214 nm)

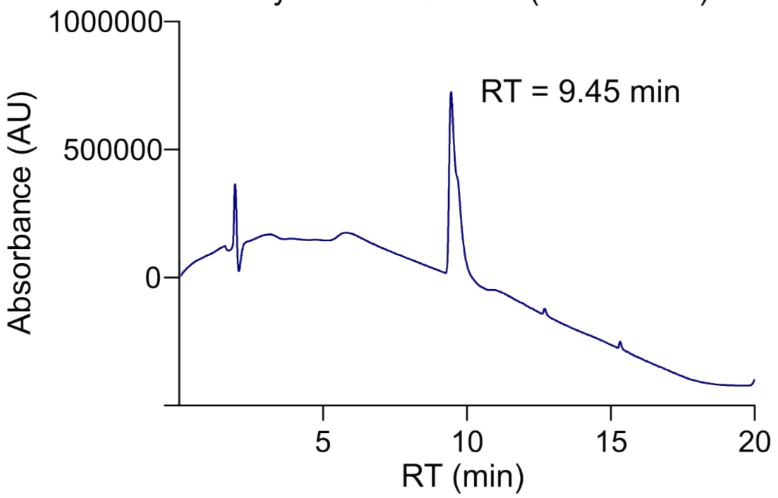

L8. Cyclo[Ac-YSHLGKKPFWTLYGPTC]G-NH_2

LC-MS: (+ESI) m/z Calculated mass for $[C_{100}H_{140}N_{24}O_{24}S + H]^+$: 2094.0227, found:$[M+2H]^{2+}$ 1047.5178, deconvoluted: 2094.04.

Analytical HPLC: Retention time (Rt) = 9.15 min (10-70 vol.% MeCN in H_2O with 0.1 vol.% FA over 20 min, λ = 214 nm).

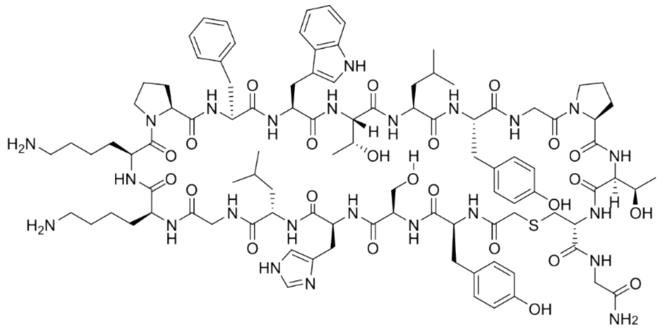

Cyclo[Ac-YSHLGKKPFWTLYGPTC]G-NH_2

Exact Mass: 2093.0193

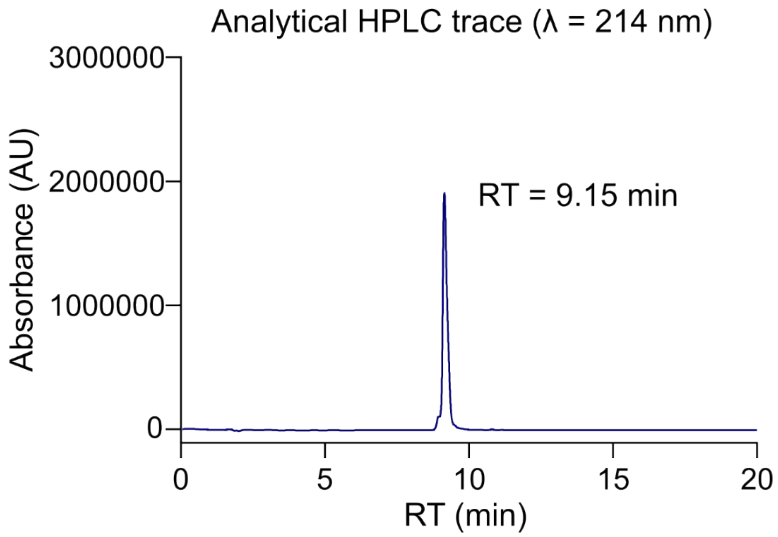

D1-1. Cyclo[Ac-yRTFWTLYGPIVPLC]SAG-NH_2

LC-MS: (+ESI) m/z Calculated mass for [$C_{100}H_{143}N_{23}O_{24}S$ +H]$^+$: 2083.0397, found:[M+2H]$^{2+}$ 1042.0292, deconvoluted: 2083.06.

Analytical HPLC: Retention time (Rt) = 8.48 min (10-70 vol.% MeCN in H_2O with 0.1 vol.% FA over 20 min, λ = 214 nm).

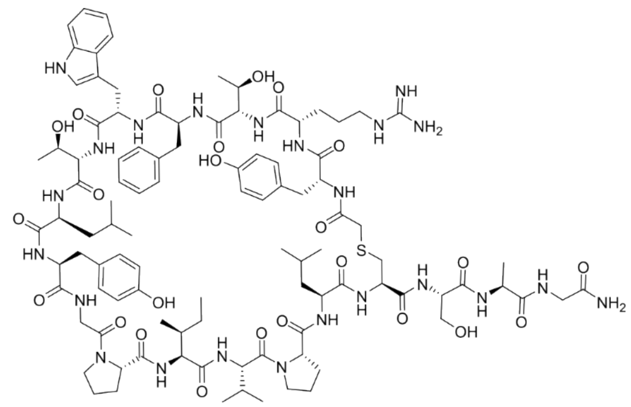

Cyclo[Ac-yRTFWTLYGPIVPLC]SAG-NH_2

Exact Mass: 2082.0397

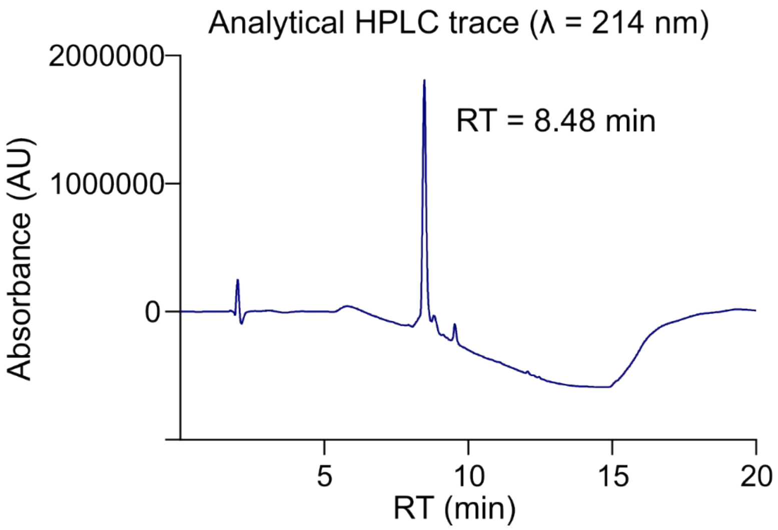

D1-2. Cyclo[Ac-yRTFWTLYGPIVPLASC]G-NH_2

LC-MS: (+ESI) m/z Calculated mass for $[C_{100}H_{143}N_{23}O_{24}S + H]^+$: 2083.0397, found:$[M+2H]^{2+}$ 1042.0279, deconvoluted: 2083.06.

Analytical HPLC: Retention time (Rt) = 8.48 min (10-70 vol.% MeCN in H_2O with 0.1 vol.% FA over 20 min, λ = 214 nm).

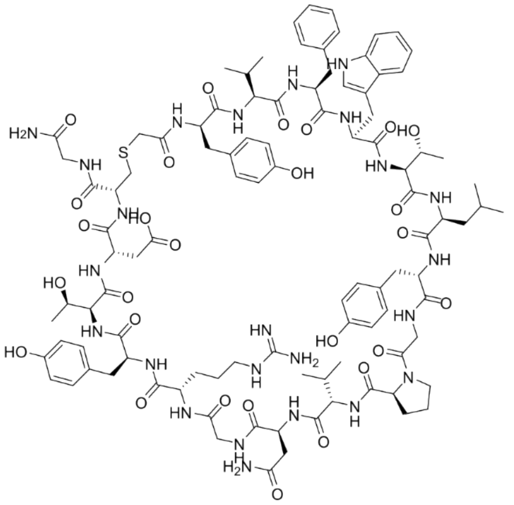

Cyclo[Ac-yRTFWTLYGPIVPLASC]G-NH_2

Exact Mass: 2148.9727

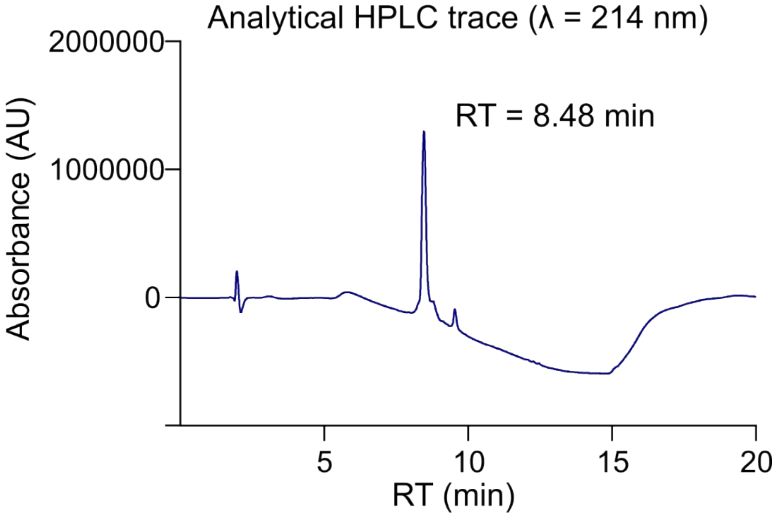

D4. Cyclo[Ac-yDYNYRRVFWSLYGPIC]G-NH_2

LC-MS: (+ESI) m/z Calculated mass for $[C_{110}H_{147}N_{27}O_{27}S + H]^+$: 2310.068, found:$[M+2H]^{2+}$ 1156.0426, deconvoluted: 2311.09.

Analytical HPLC: Retention time (Rt) = 10.07 min (10-70 vol.% MeCN in H_2O with 0.1 vol.% FA over 20 min, λ = 214 nm).

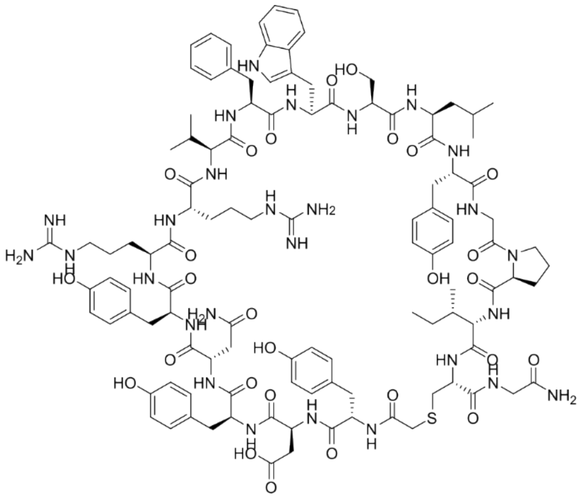

Cyclo[Ac-yDYNTRRVFWSLYGPIC]G-NH_2

Exact Mass: 2310.0680

Analytical HPLC trace (λ = 214 nm)

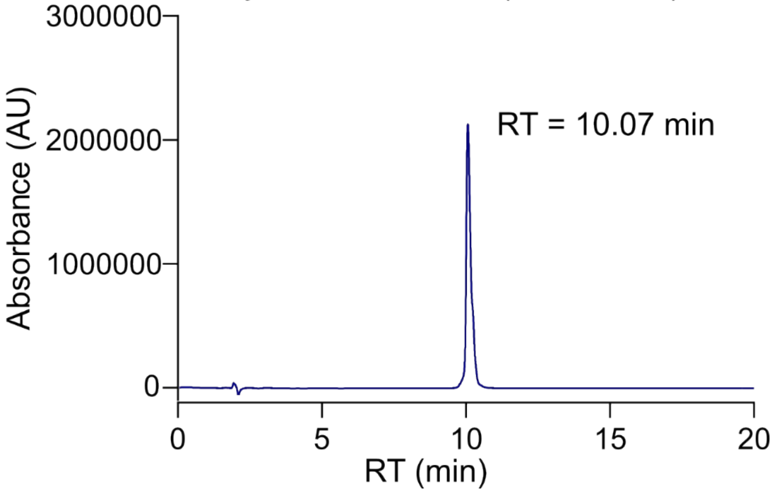

D7. Cyclo[Ac-yRSQYVDRFLYRVPIDC]G-NH_2

LC-MS: (+ESI) m/z Calculated mass for $[C_{102}H_{149}N_{29}O_{29}S+H]^+$: 2310.068, found:$[M+2H]^{2+}$ 1156.0426, deconvoluted: 2311.09.

Analytical HPLC: Retention time (Rt) = 8.76 min (10-70 vol.% MeCN in H_2O with 0.1 vol.% FA over 20 min, λ = 214 nm).

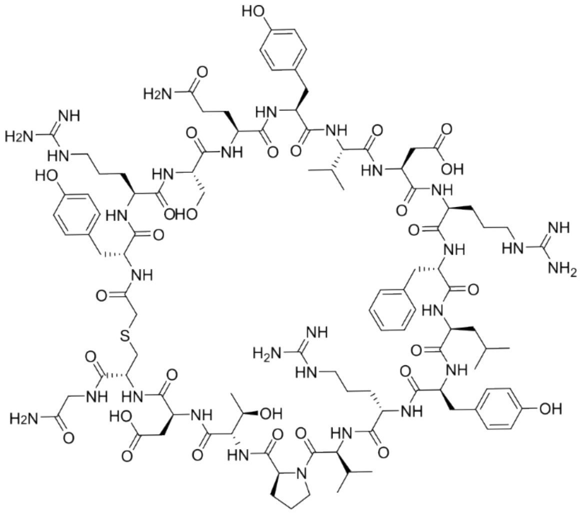

Cyclo[Ac-yRSQYVDRFLYRVPIDC]G-NH_2

Exact Mass: 2276.0797

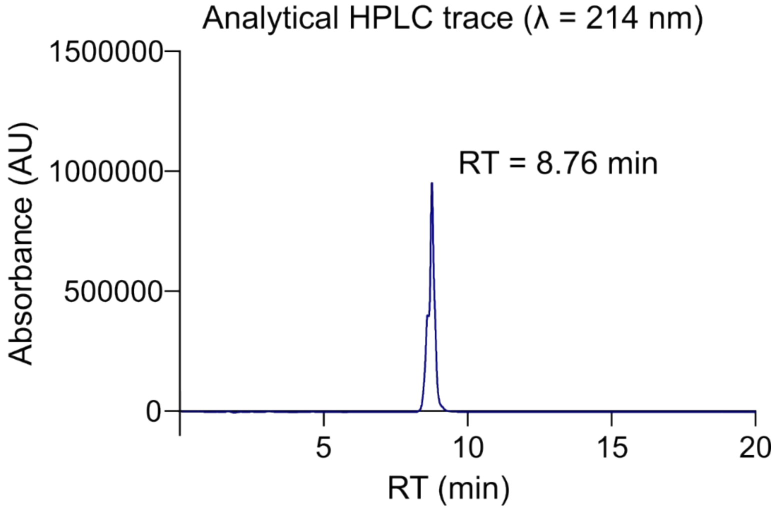

PABP1[456-466]. Ac-PAAPRPPFSTM-NH_2

LC-MS: (+ESI) m/z Calculated mass for $[C_{55}H_{85}N_{15}O_{14}S +H]^+$: 1212.61, found: 1213.5 $[M+H]^+$, 607.0 $[M+2H]^{2+}$, deconvoluted: 1213.0.

Analytical HPLC: Retention time (Rt) = 16.34 min (10-70 vol.% MeCN in H_2O with 0.1 vol.% FA over 50 min, λ = 215 nm).

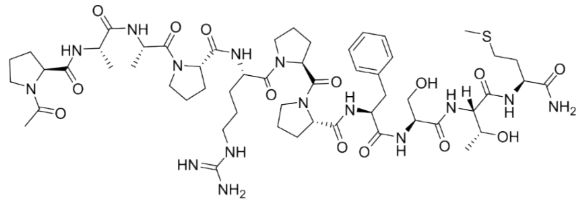

Ac-PAAPRPPFSTM-NH_2

Exact Mass: 1211.6121

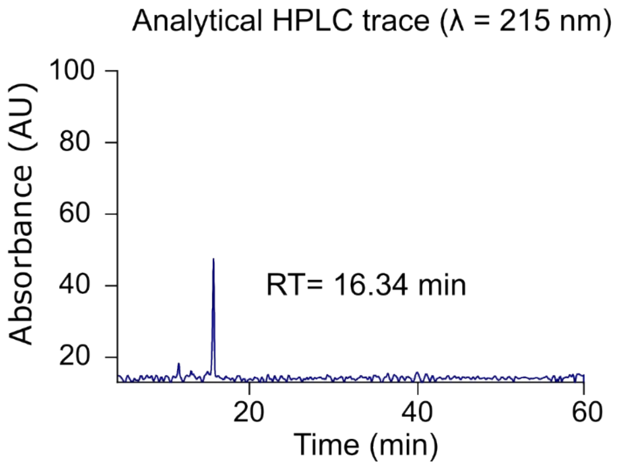

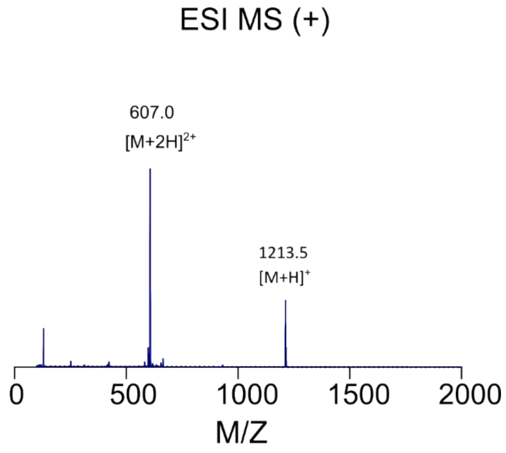

L63-69. Cyclo [Ac-YFHGFNRC] LYPPRYDIAG-NH_2
LC-MS: (+ESI) m/z Calculated mass for [$C_{105}H_{141}N_{27}O_{26}S$ +H]$^+$: 2229.03, found: 743.7 [M+3H]$^{3+}$, 1114.7 [M+2H]$^{2+}$, deconvoluted: 2229.10.
Analytical HPLC: Retention time (Rt) = 16.72 min (10-70 vol.% MeCN in H_2O with 0.1 vol.% FA over 50 min, λ = 215 nm).

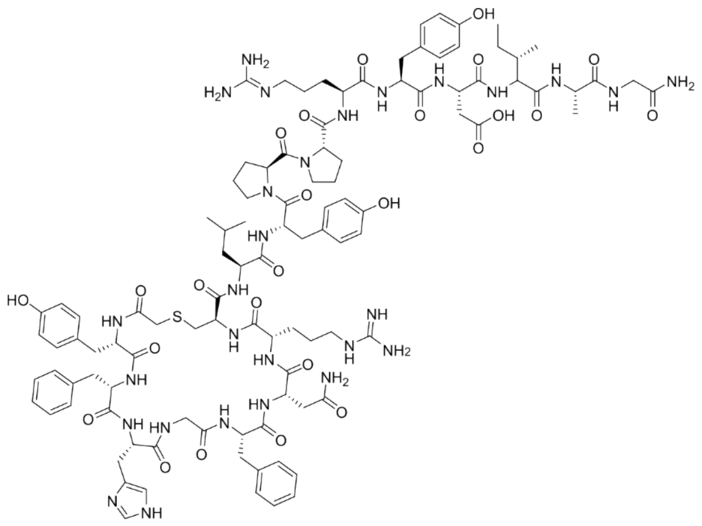

Ccyclo[Ac-YFHGFNRC]LYPPRYDIAG-NH_2
Exact Mass: 2227.0422

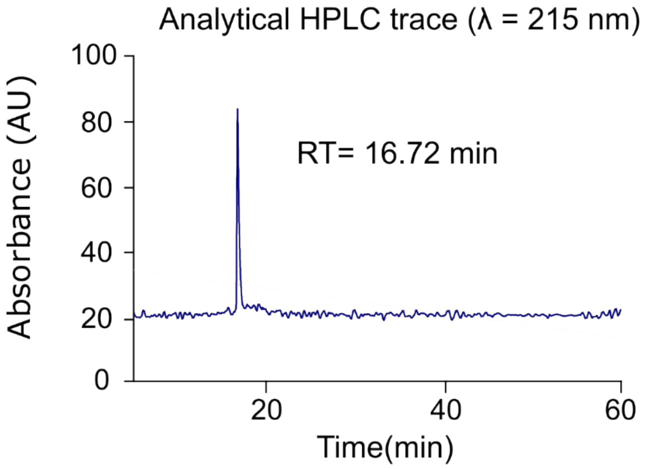

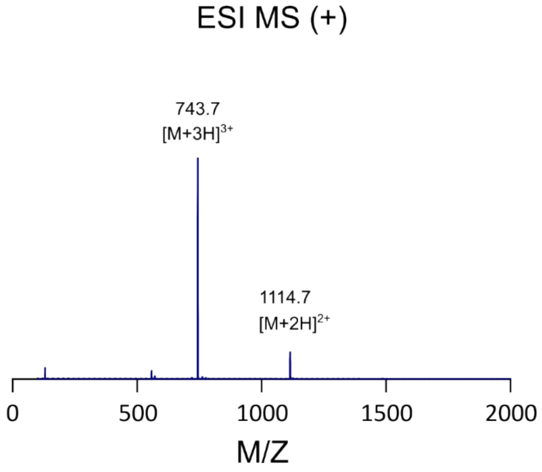

L171-772. Cyclo [Ac-YWKDFIRC]IYRPPIIDAG-NH_2

LC-MS: (+ESI) m/z Calculated mass for [$C_{108}H_{157}N_{27}O_{25}S$ +H]$^+$: 2265.16, found: 756.10 [M+3H]$^{3+}$, 1133.5 [M+2H]$^{2+}$, deconvoluted: 2265.30.

Analytical HPLC: Retention time (Rt) = 21.70 min (10-70 vol.% MeCN in H_2O with 0.1 vol.% FA over 50 min, λ = 215 nm).

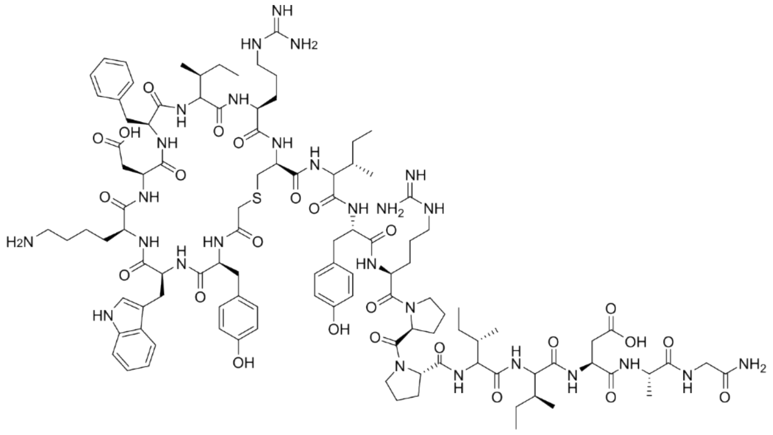

Cyclo[Ac-YWKDFIRC]IYRPPIIDAG-NH_2

Exact Mass: 2264.1565

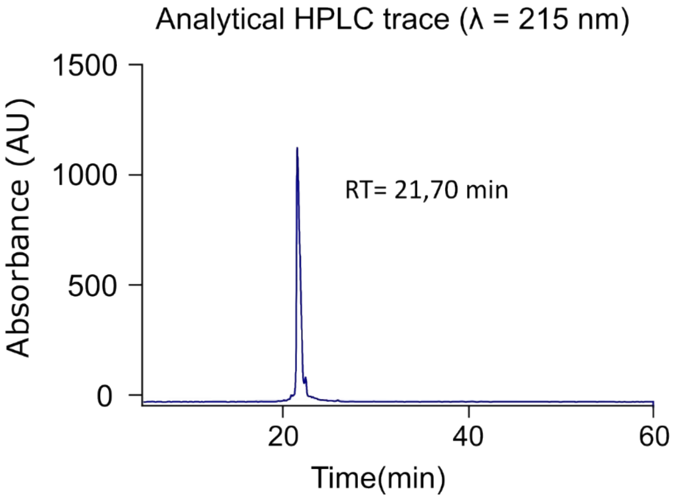

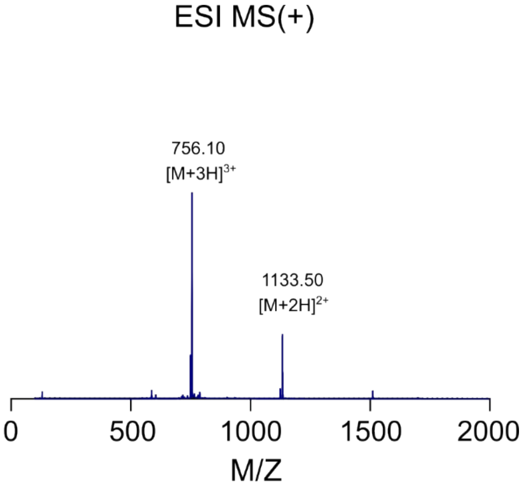

L21-34. Cyclo[Ac-YFHFTWPRFPTPRGLWC]G-NH_2
LC-MS: (+ESI) m/z Calculated mass for $[C_{114}H_{147}N_{29}O_{22}S +H]^{+}$: 2307.1, found: 770.10 $[M+3H]^{3+}$, 1154.6 $[M+2H]^{2+}$, deconvoluted: 2307.27.
Analytical HPLC: Retention time (Rt) = 23.23 min (10-70 vol.% MeCN in H_2O with 0.1 vol.% FA over 50 min, λ = 215 nm).

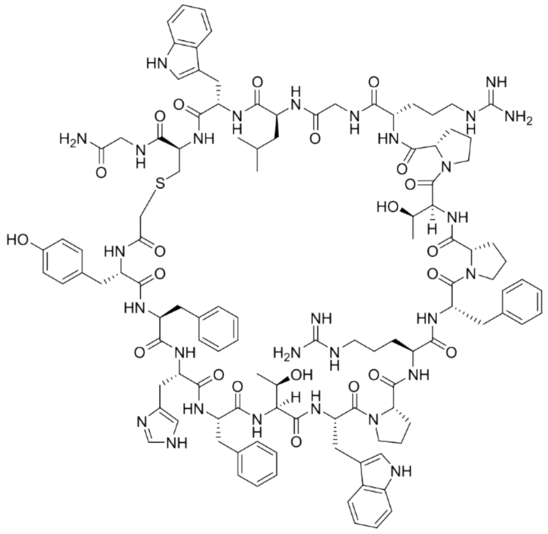

Cyclo[Ac-YFHFTWPRFPTPRGLWC]G-NH_2
Exact Mass: 2306.0996

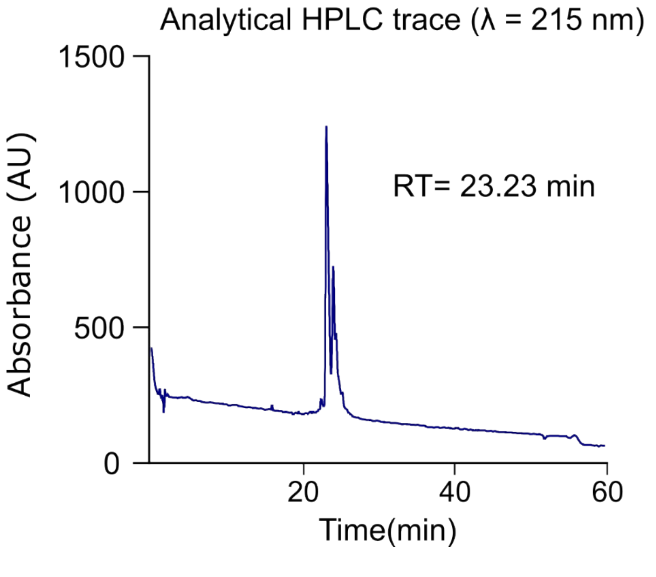

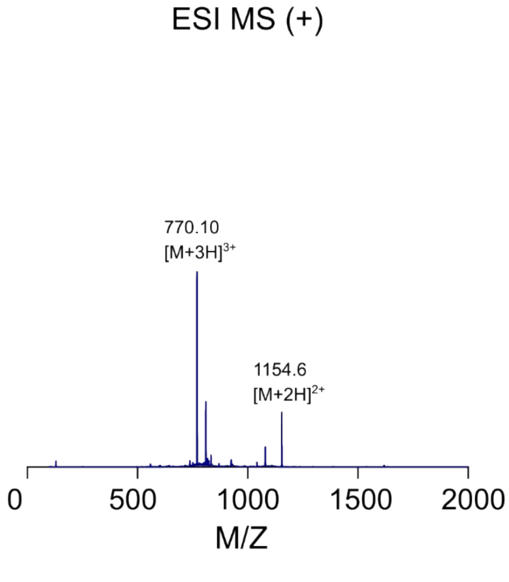

D28-36. Cyclo[Ac-yFWFTRPC]ALS[CARTYC]G-NH_2

LC-MS: (+ESI) m/z Calculated mass for $[C_{100}H_{138}N_{26}O_{24}S_3 + H]^+$: 2183.95, found: 728.8 $[M+3H]^{3+}$, 1092.6 $[M+2H]^{2+}$, deconvoluted: 2184.2.

Analytical HPLC: Retention time (Rt) = 25.59 min (10-70 vol.% MeCN in H_2O with 0.1 vol.% FA over 50 min, λ = 215 nm).

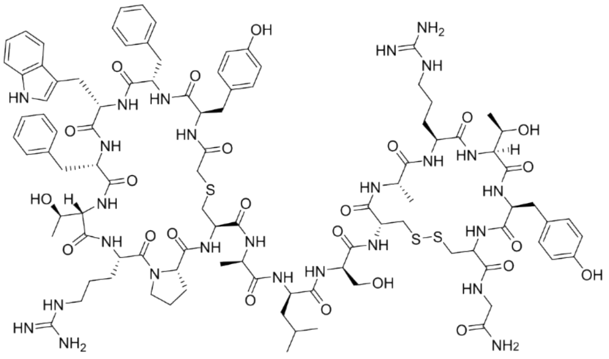

Cyclo[Ac-yFWFTRPC]ALS[CARTYC]G-NH_2

Exact Mass: 2180.94

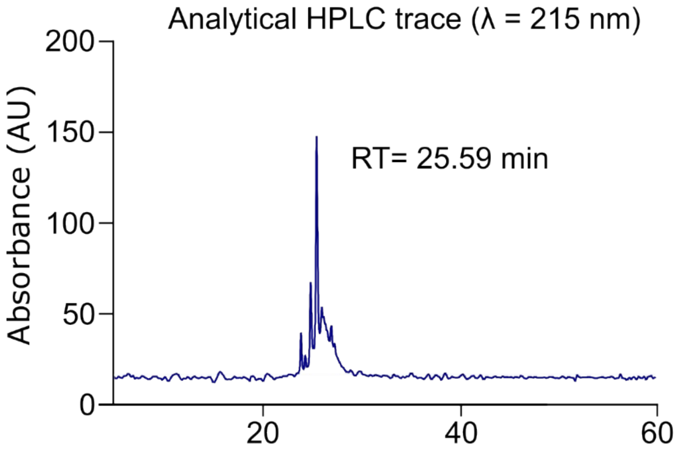

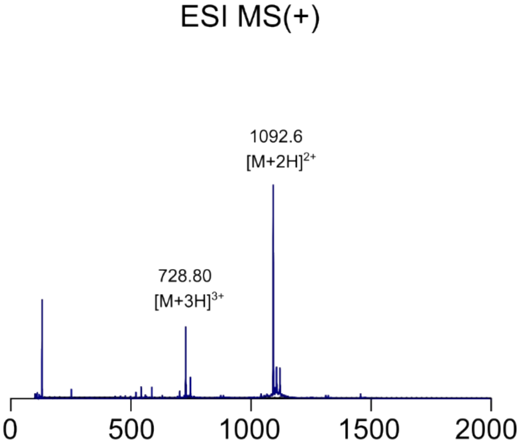

D51-54. Cyclo[Ac-yTWYYQAAYC]KLPSVPAG-NH_2

LC-MS: (+ESI) m/z Calculated mass for $[C_{102}H_{137}N_{21}O_{27}S + H]^+$: 2120.97, found: 2122.8 $[M+3H]^{3+}$, 2122.2 $[M+2H]^{2+}$, deconvoluted: 2121.2.

Analytical HPLC: Retention time (Rt) = 22.53 min (10-70 vol.% MeCN in H_2O with 0.1 vol.% FA over 50 min, λ = 215 nm).

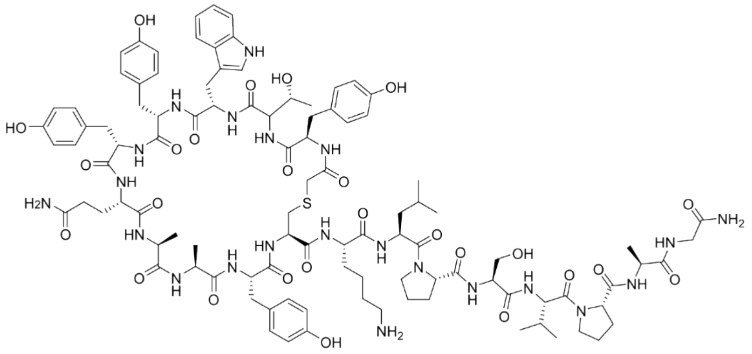

Cyclo[Ac-yTWYYQAAYC]KLPSVPAG-NH_2

Exact Mass: 2119.97

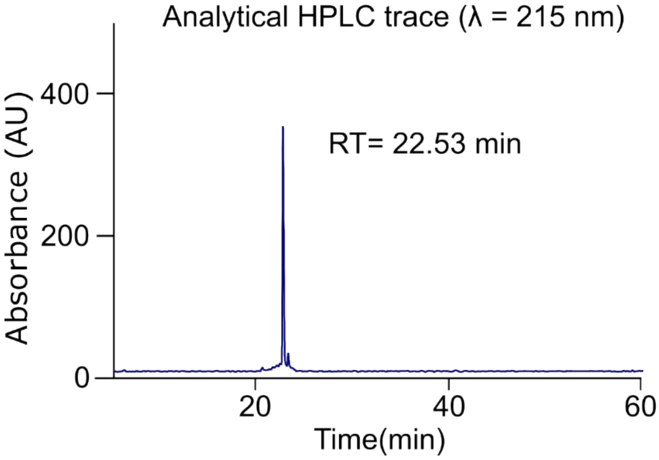

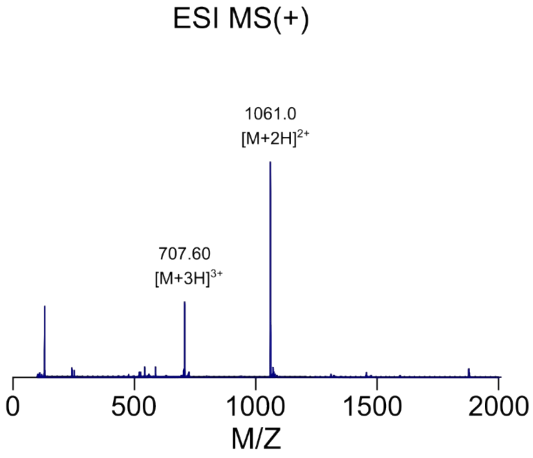

D59-129. Cyclo[Ac-yWYWIRFPTPKFQTLKC]G-NH_2

LC-MS: (+ESI) m/z Calculated mass for [$C_{118}H_{161}N_{27}O_{24}S$ +H]$^+$: 2373.19, found: 792.10 [M+3H]$^{3+}$, 1187.6 [M+2H]$^{2+}$, deconvoluted: 2373.3.

Analytical HPLC: Retention time (Rt) = 21.67 min (10-70 vol.% MeCN in H_2O with 0.1 vol.% FA over 50 min, λ = 215 nm).

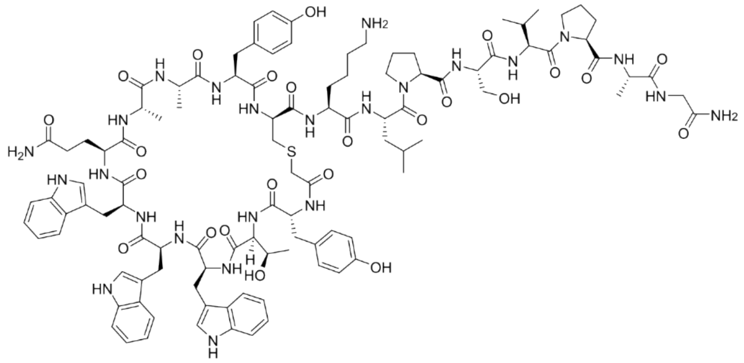

Cyclo[Ac-yWYWIRFPTPKFQTLKC]G-NH_2

Exact Mass: 2165.02

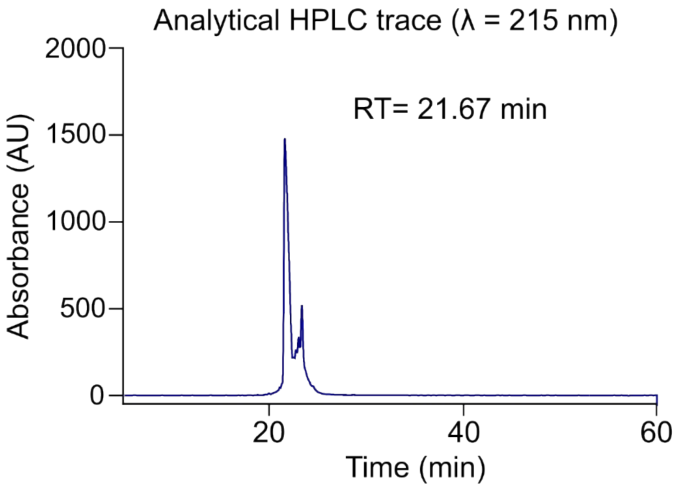

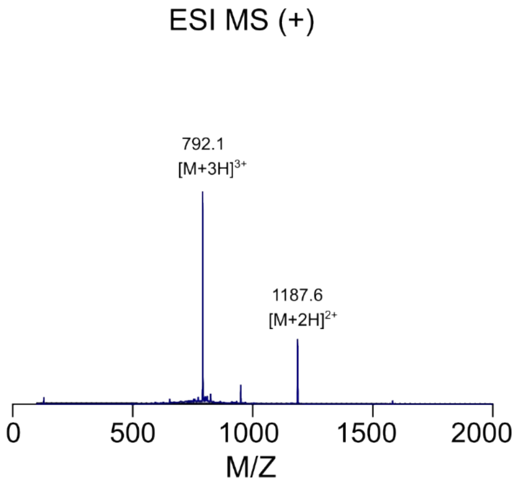

L171-772_cyclic.

LC-MS: (+ESI) m/z Calculated mass for [$C_{56}H_{76}N_{14}O_{12}S$ +H]$^+$: 1169.55, found: 585.4 [M+2H]$^{2+}$, deconvoluted: 1169.8.

Analytical HPLC: Retention time (Rt) = 18.35 min (10-70 vol.% MeCN in H_2O with 0.1 vol.% FA over 50 min, λ = 215 nm).

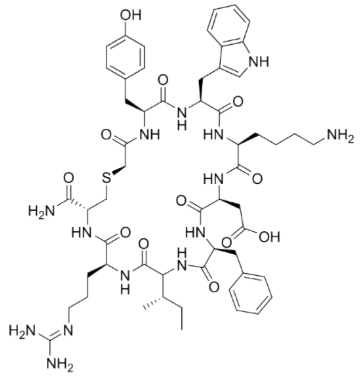

Cyclo[Ac-YWKDFIRC]-NH_2

Exact Mass: 1168.55

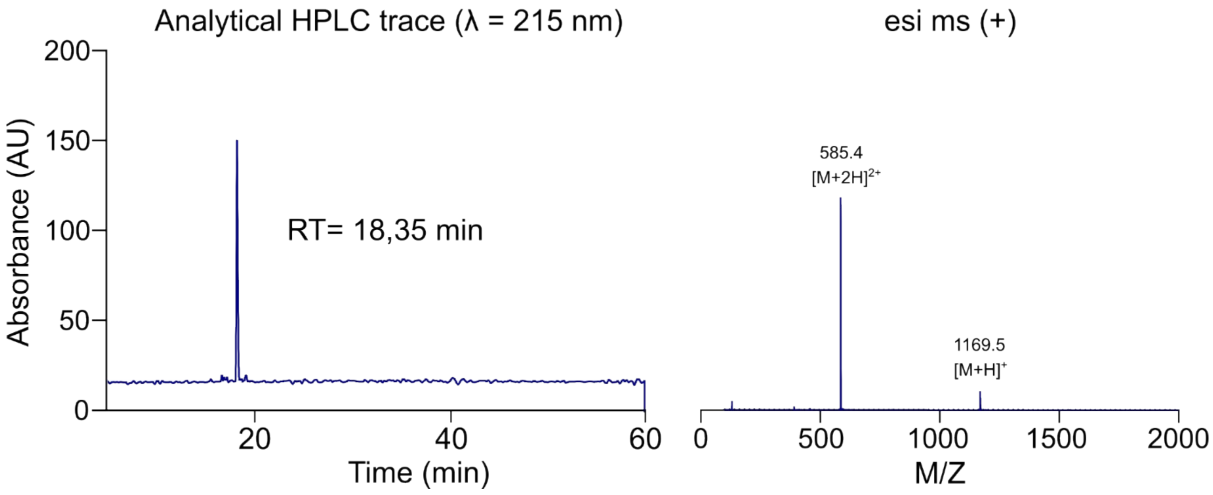

L171-772_tail.

LC-MS: (+ESI) m/z Calculated mass for $[C_{54}H_{86}N_{14}O_{14}S + H]^+$: 1155.64, found: 1155.6 $[M+H]^+$, 578.5 $[M+2H]^{2+}$, deconvoluted: 1155.6.

Analytical HPLC: Retention time (Rt) = 19.77 min (10-70 vol.% MeCN in H_2O with 0.1 vol.% FA over 50 min, λ = 215 nm).

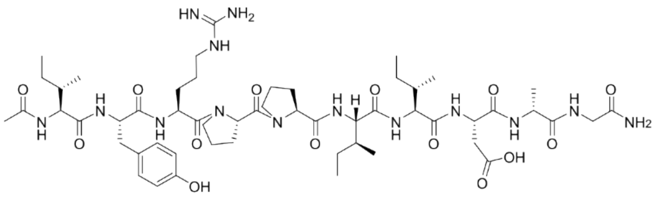

Ac-IYRPPIIDAG-NH_2

Exact Mass: 1154.64

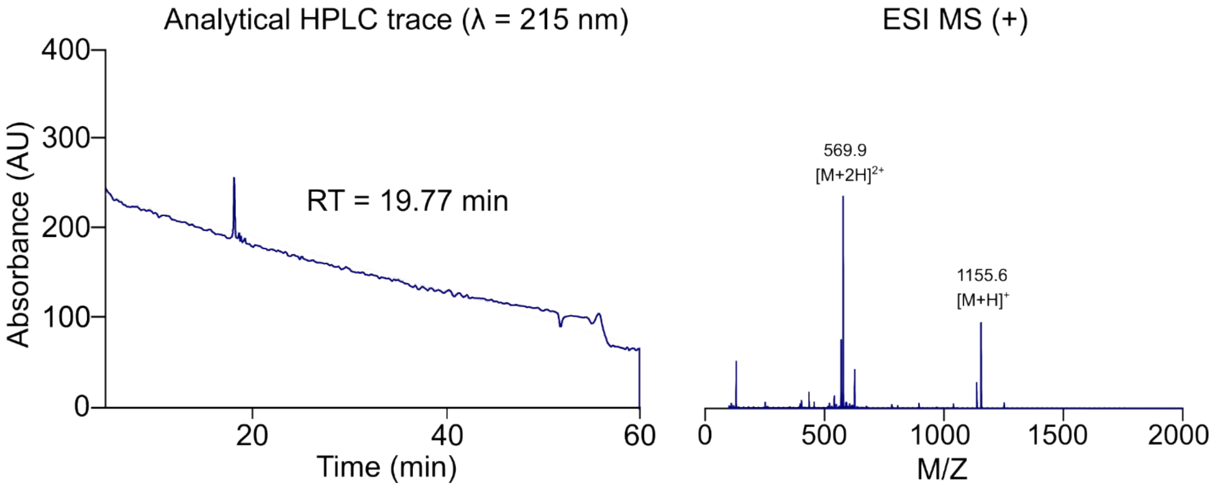

L171-772-R7K. Cyclo[Ac-YWKDFIYC] IYRPPIIDAG-NH_2

LC-MS: (+ESI) m/z Calculated mass for $[C_{108}H_{157}N_{25}O_{25}S + H]^+$: 2237.15, found: 1119.5 $[M+2H]^{2+}$, 746.7 $[M+3H]^{3+}$, deconvoluted: 2238.0.

Analytical HPLC: Retention time (Rt) = 13.75 min (10-70 vol.% MeCN in H_2O with 0.1 vol.% FA over 50 min, λ = 215 nm).

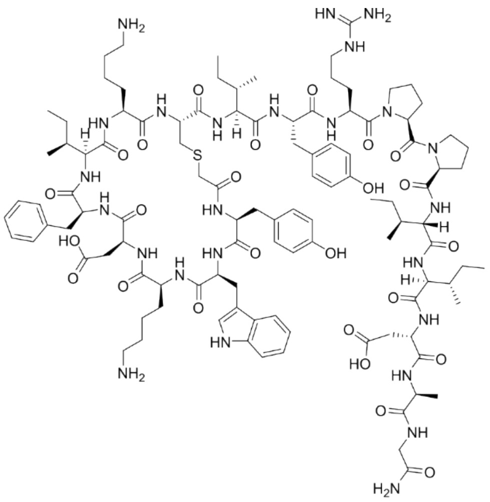

Cyclo[Ac-YWKDFIKC]IYRPPIIDAG-NH_2

Exact Mass: 2236.15

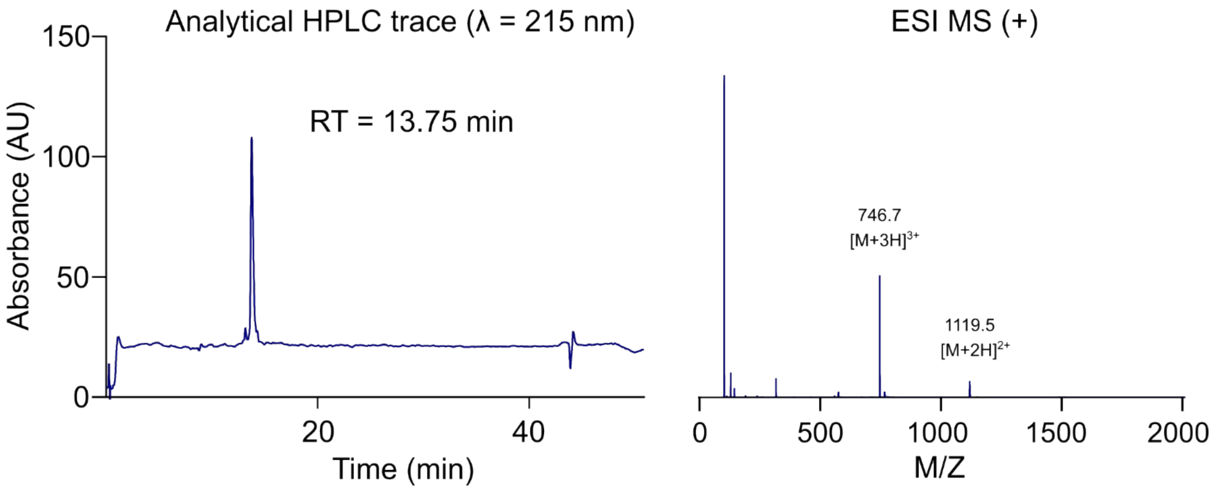

L171-772-R11K. Cyclo[Ac-YWKDFIRC] IYYPPIIDAG-NH_2

LC-MS: (+ESI) m/z Calculated mass for $[C_{108}H_{157}N_{25}O_{25}S +H]^+$: 2237.15, found: 1119.5 $[M+2H]^{2+}$, 746.7 $[M+3H]^{3+}$, deconvoluted: 2238.0.

Analytical HPLC: Retention time (Rt) = 13.68 min (10-70 vol.% MeCN in H_2O with 0.1 vol.% FA over 50 min, λ = 215 nm).

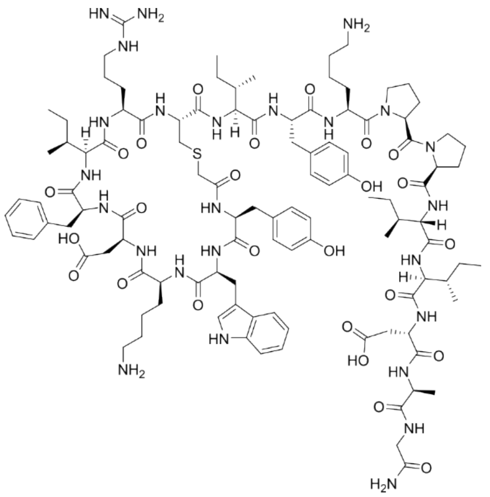

Cyclo[Ac-YWKDFIRC]IYKPPIIDAG-NH_2

Exact Mass: 2236.15

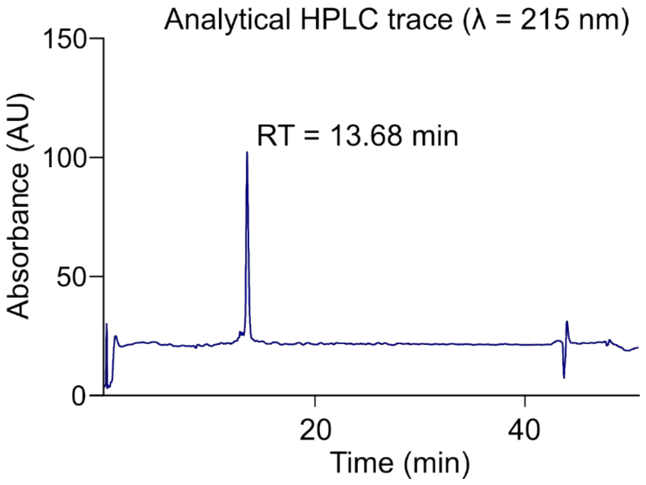

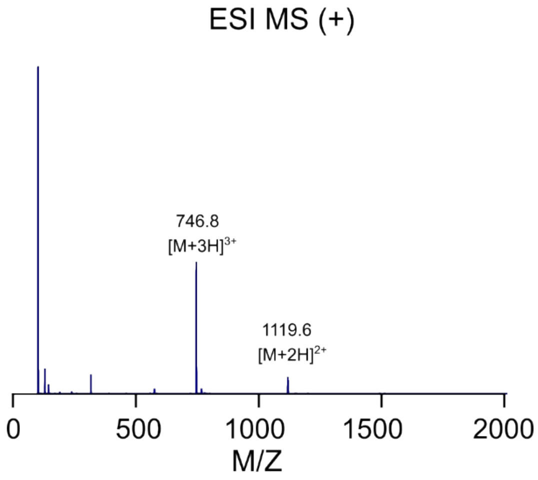

L171-772_Cit. Cyclo[Ac-YWKDFIxC]IYxPPIIDAG-NH2 x=Citrulline
LC-MS: (+ESI) m/z Calculated mass for $[C_{108}H_{155}N_{25}O_{27}S +H]^+$: 2266.12, found: 1134.20 $[M+2H]^{2+}$, 756.7 $[M+3H]^{3+}$, deconvoluted: 2266.40.
Analytical HPLC: Retention time (Rt) = 27.00 min (10-70 vol.% MeCN in H_2O with 0.1 vol.% FA over 50 min, λ = 215 nm).

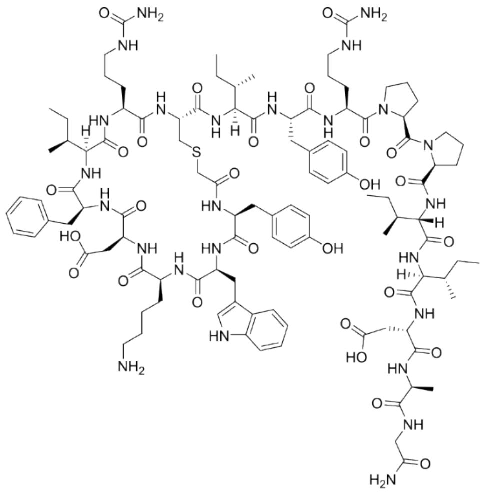

Cyclo[Ac-YWKDFIxC]IYxPPIIDAG-NH_2 x=Citrulline

Exact Mass: 2266.12

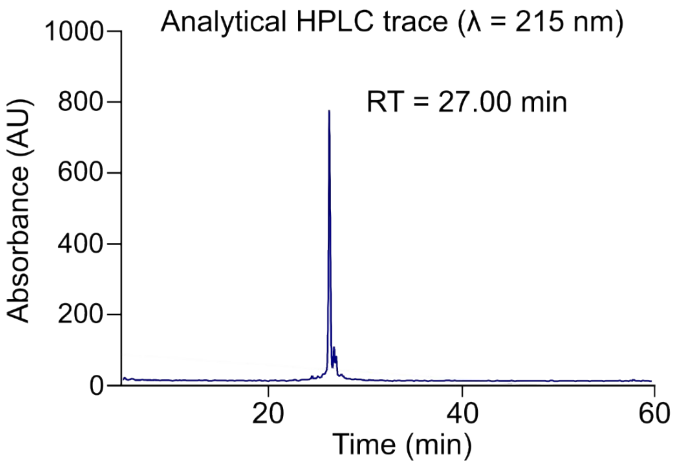

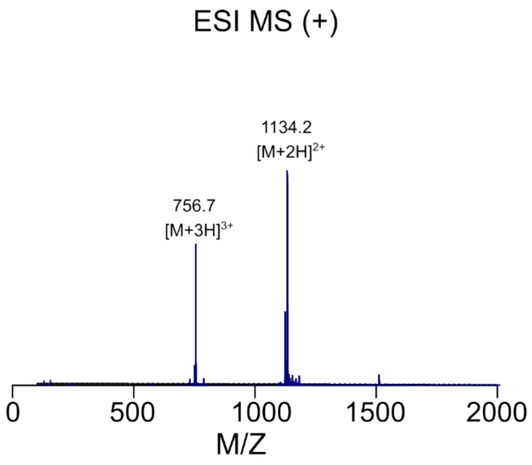

L171-772_R(Me)$_2$. Cyclo[Ac-YWKDFIrC]IYrPPIIDAG-NH$_2$ r=demethylated arginine

LC-MS: (+ESI) m/z Calculated mass for [$C_{112}H_{165}N_{27}O_{25}S$ +H]$^+$: 2320.22, found: 1161.30 [M+2H]$^{2+}$, 774.7 [M+3H]$^{3+}$, deconvoluted: 2320.6.

Analytical HPLC: Retention time (Rt) = 23.75 min (10-70 vol.% MeCN in H_2O with 0.1 vol.% FA over 50 min, λ = 215 nm).

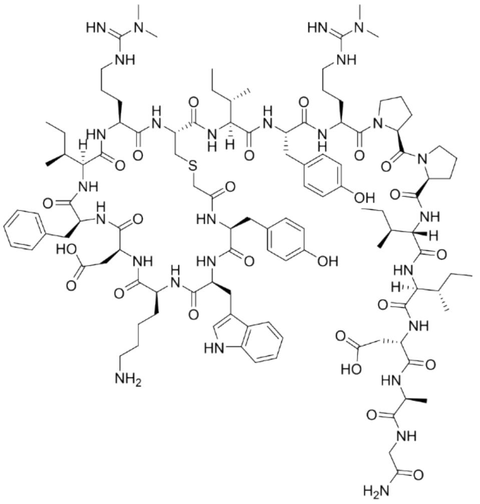

Cyclo[Ac-YWKDFIrC]IYrPPIIDAG-NH$_2$ r=di-methylated arginine

Exact Mass: 2320.22

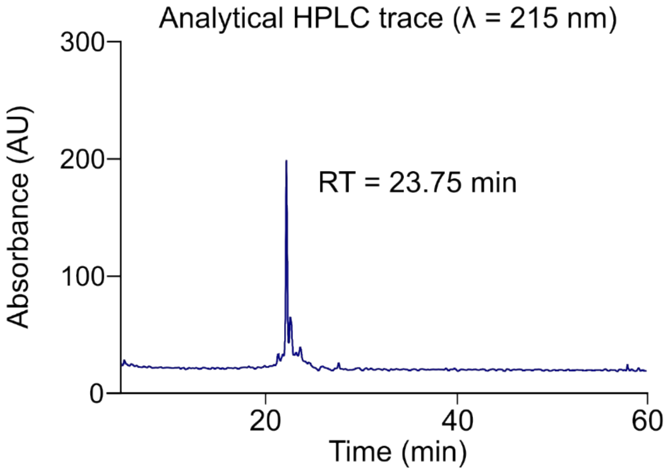

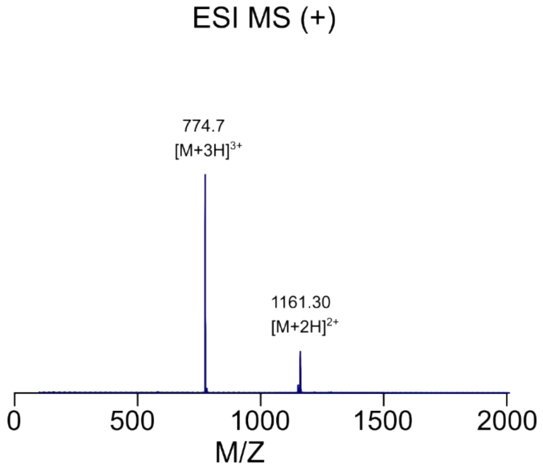

D28-36-cyclic. cyclo[Ac-yFWFTRPC]-NH_2
LC-MS: (+ESI) m/z Calculated mass for $[C_{58}H_{71}N_{13}O_{11}S + H]^+$: 1158.51, found: 1158.40 $[M+H]^+$, 579.90 $[M+2H]^{2+}$, deconvoluted: 1158.40.
Analytical HPLC: Retention time (Rt) = 13.84 min (10-70 vol.% MeCN in H_2O with 0.1 vol.% FA over 50 min, λ = 215 nm).

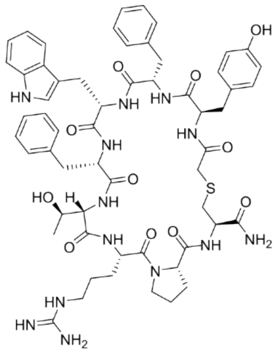

Cyclo[Ac-yFWFTRPC]-NH_2
Exact Mass: 1157.51

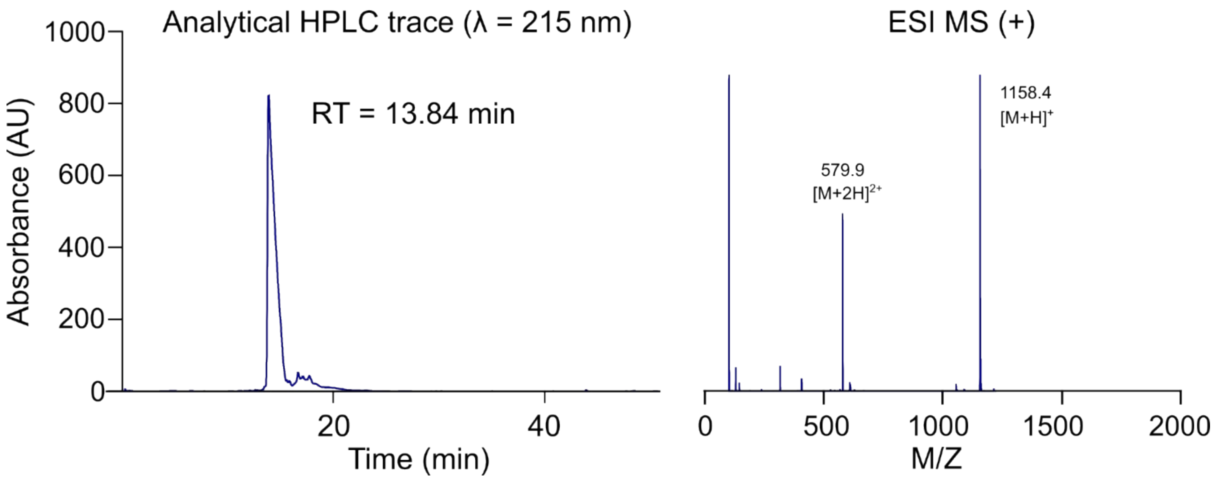

L1-FAM. Cyclo[Ac- YSHLGKKPFWTLYGPIC]GppYfG-NH_2

LC-MS: (+ESI) m/z Calculated mass for $[C_{171}H_{216}N_{34}O_{40}S + H]^+$: 3418.5707, found: 1710.8 $[M+2H]^{2+}$, 1140.6 $[M+3H]^{3+}$, 855.8 $[M+4H]^{4+}$, deconvoluted: 3418.8.

Analytical HPLC: Retention time (Rt) = 55.5 min (10-70 vol.% MeCN in H_2O with 0.1 vol.% FA over 60 min, λ = 280 nm).

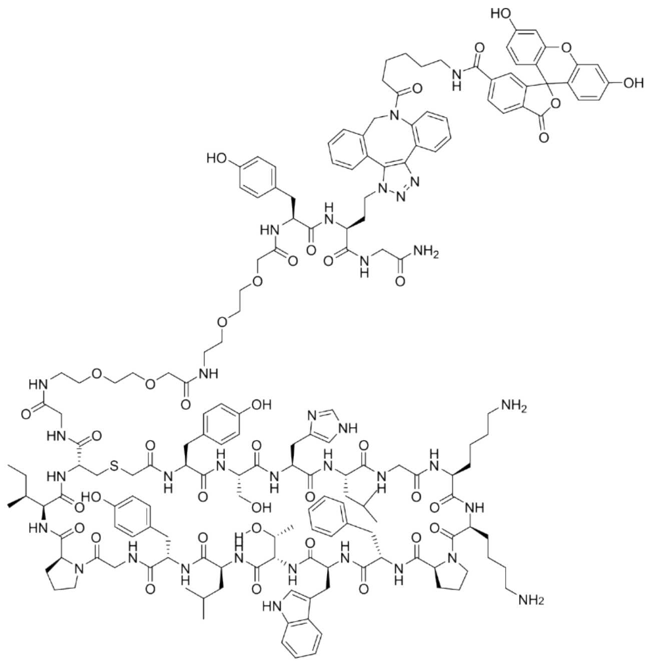

Cyclo[Ac-YSHLGKKPFWTLYGPIC]GppYfG-NH_2

Exact Mass: 3417.562

Analytical HPLC trace (λ = 280 nm)

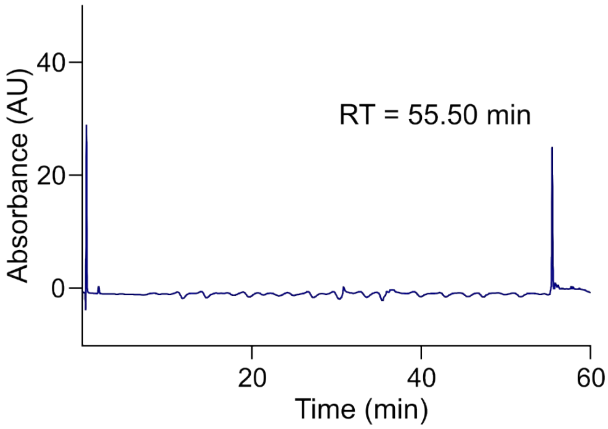

ESI(+)

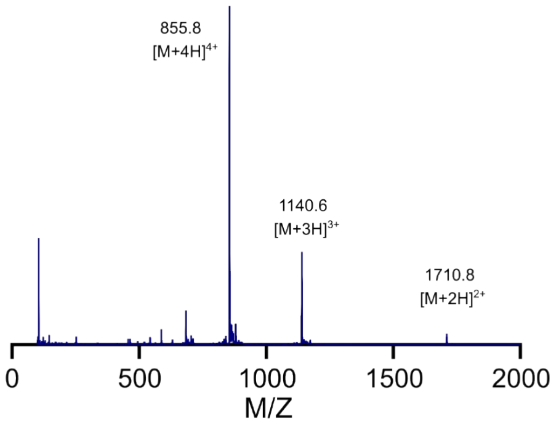

Supplementary references

[1] Y. Zhang, M. J. van Haren, N. Marechal, N. Troffer-Charlier, V. Cura, J. Cavarelli, N. I. Martin, “A Direct Assay for Measuring the Activity and Inhibition of Coactivator-Associated Arginine Methyltransferase 1” *Biochemistry* 2022, *61*, 1055–1063.

[2] M. J. Van Haren, Y. Zhang, V. Thijssen, N. Buijs, Y. Gao, L. Mateuszuk, F. A. Fedak, A. Kij, R. Campagna, D. Sartini, “Macrocyclic peptides as allosteric inhibitors of nicotinamide N-methyltransferase (NNMT)” *RSC Chem. Biol.* 2021, *2*, 1546–1555.

[3] W. Li, A. Godzik, “Cd-hit: a fast program for clustering and comparing large sets of protein or nucleotide sequences” *Bioinformatics* 2006, *22*, 1658–1659.

[4] W. Li, L. Jaroszewski, A. Godzik, “Clustering of highly homologous sequences to reduce the size of large protein databases” *Bioinformatics* 2001, *17*, 282–283.

[5] W. Li, L. Jaroszewski, A. Godzik, “Tolerating some redundancy significantly speeds up clustering of large protein databases” *Bioinformatics* 2002, *18*, 77–82.

[6] H. Berberich, F. Terwesten, S. Rakow, P. Sahu, C. Bouchard, M. Meixner, S. Philipsen, P. Kolb, U.-M. Bauer, “Identification and in silico structural analysis of Gallus gallus protein arginine methyltransferase 4 (PRMT4)” *FEBS Open Bio* 2017, *7*, 1909–1923.

[7] A. Repenning, D. Happel, C. Bouchard, M. Meixner, Y. Verel-Yilmaz, H. Raifer, L. Holembowski, E. Krause, E. Kremmer, R. Feederle, C. U. Keber, M. Lohoff, E. P. Slater, D. K. Bartsch, U. Bauer, “PRMT1 promotes the tumor suppressor function of p14ARF and is indicative for pancreatic cancer prognosis” *EMBO J.* 2021, *40*, e106777.

[8] H. Naeem, D. Cheng, Q. Zhao, C. Underhill, M. Tini, M. T. Bedford, J. Torchia, “The Activity and Stability of the Transcriptional Coactivator p/CIP/SRC-3 are Regulated by CARM1-Dependent Methylation” *Mol. Cell. Biol.* 2007, *27*, 120–134.

[9] K. Bouazoune, R. E. Kingston, “Chromatin remodeling by the CHD7 protein is impaired by mutations that cause human developmental disorders” *Proceedings of the National Academy of Sciences* 2012, *109*, 19238–19243.

[10] R. V Hosur, G. Wider, K. Wüthrich, “Sequential Individual Resonance Assignments in the 1H Nuclear-Magnetic-Resonance Spectrum of Cardiotoxin VII 2 from Naju mossambica rnossumbica” *Eur. J. Biochem.* 1981, *130*, 497–508.

[11] T. Harsch, P. Schneider, B. Kieninger, H. Donaubauer, H. R. Kalbitzer, “Stereospecific assignment of the asparagine and glutamine sidechain amide protons in proteins from chemical shift analysis” *J. Biomol. NMR* 2017, *67*, 157–164.

[12] W. Lee, M. Rahimi, Y. Lee, A. Chiu, “POKY: a software suite for multidimensional NMR and 3D structure calculation of biomolecules” *Bioinformatics* 2021, *37*, 3041–3042.

[13] T. Aeschbacher, M. Schubert, F. H.-T. Allain, “A procedure to validate and correct the 13C chemical shift calibration of RNA datasets” *J. Biomol. NMR* 2012, *52*, 179–190.

[14] Y. Shen, F. Delaglio, G. Cornilescu, A. Bax, “TALOS+: a hybrid method for predicting protein backbone torsion angles from NMR chemical shifts.” *J. Biomol. NMR* 2009, *44*, 213–223.

[15] P. Güntert, L. Buchner, “Combined automated NOE assignment and structure calculation with CYANA” *J. Biomol. NMR* 2015, *62*, 453–471.

[16] M. Schubert, D. Labudde, H. Oschkinat, P. Schmieder, “A software tool for the prediction of Xaa-Pro peptide bond conformations in proteins based on 13 C chemical shift statistics” *J. Biomol. NMR* 2002, *24*, 149–154.

[17] P. A. Boriack-Sjodin, L. Jin, S. L. Jacques, A. Drew, C. Sneeringer, M. P. Scott, M. P. Moyer, S. Ribich, O. Moradei, R. A. Copeland, “Structural insights into ternary complex formation of human CARM1 with various substrates” *ACS Chem. Biol.* 2016, *11*, 763–771.

[18] D. A. Case, H. M. Aktulga, K. Belfon, D. S. Cerutti, G. A. Cisneros, V. W. D. Cruzeiro, N. Forouzesh, T. J. Giese, A. W. Götz, H. Gohlke, S. Izadi, K. Kasavajhala, M. C. Kaymak, E. King, T. Kurtzman, T.-S. Lee, P. Li, J. Liu, T. Luchko, R. Luo, M. Manathunga, M. R. Machado, H. M. Nguyen, K. A. O'Hearn, A. V Onufriev, F. Pan, S. Pantano, R. Qi, A. Rahnamoun, A. Risheh, S. Schott-Verdugo, A. Shajan, J. Swails, J. Wang, H. Wei, X. Wu, Y. Wu, S. Zhang, S. Zhao, Q. Zhu, T. E. I. I. I. Cheatham, D. R. Roe, A. Roitberg, C. Simmerling, D. M. York, M. C. Nagan, K. M. Jr. Merz, "AmberTools" *J. Chem. Inf. Model.* 2023, *63*, 6183–6191.

[19] D. A. Case, H. M. Aktulga, K. Belfon, I. Y. Ben-Shalom, J. T. Berryman, S. R. Brozell, F. S. Carvahol, D. S. Cerutti, T. E. Cheatham III, G. A. Cisneros, V. W. D. Cruzeiro, T. A. Darden, N. Forouzesh, M. Ghazimirsaeed, G. Giambaşu, T. Giese, M. K. Gilson, H. Gohlke, A. W. Goetz, J. Harris, Z. Huang, S. Izadi, S. A. Izmailov, K. Kasavajhala, M. C. Kaymak, I. Kolossváry, A. Kovalenko, T. Kurtzman, T. S. Lee, P. Li, Z. Li, C. Lin, J. Liu, T. Luchko, R. Luo, M. Machado, K. M. Manathunga, Y. Merz, O. Miao, G. Mikhailovskii, H. Monard, K. A. Nguyen, A. O'Hearn, A. Onufriev, F. Pan, S. Pantano, A. Rahnamoun, D. R. Roe, C. Roitberg, C. Sagui, S. Schott-Verdugo, A. Shajan, J. Shen, C. L. Simmerling, N. R. Skrynnikov, J. Smith, J. Swails, R. C. Walker, J. Wang, X. Wang, Y. Wu, Y. Wu, Y. Xiong, D. Xue, D. M. York, C. Zhao, Q. Zhu, P. A. Kollman, *Amber 2025*, University Of California, San Francisco, 2025.

[20] E. C. Meng, T. D. Goddard, E. F. Pettersen, G. S. Couch, Z. J. Pearson, J. H. Morris, T. E. Ferrin, "UCSF ChimeraX: Tools for structure building and analysis" *Protein Science* 2023, *32*, e4792.

[21] E. F. Pettersen, T. D. Goddard, C. C. Huang, E. C. Meng, G. S. Couch, T. I. Croll, J. H. Morris, T. E. Ferrin, "UCSF ChimeraX: Structure visualization for researchers, educators, and developers" *Protein science* 2021, *30*, 70–82.

[22] T. D. Goddard, C. C. Huang, E. C. Meng, E. F. Pettersen, G. S. Couch, J. H. Morris, T. E. Ferrin, "UCSF ChimeraX: Meeting modern challenges in visualization and analysis" *Protein science* 2018, *27*, 14–25.

[23] A. T. McNutt, P. Francoeur, R. Aggarwal, T. Masuda, R. Meli, M. Ragoza, J. Sunseri, D. R. Koes, "GNINA 1.0: molecular docking with deep learning" *J. Cheminform.* 2021, *13*, 43.

[24] R. Quiroga, M. A. Villarreal, "Vinardo: A scoring function based on autodock vina improves scoring, docking, and virtual screening" *PLoS One* 2016, *11*, e0155183.

[25] C. W. Hopkins, S. Le Grand, R. C. Walker, A. E. Roitberg, "Long-time-step molecular dynamics through hydrogen mass repartitioning" *J. Chem. Theory Comput.* 2015, *11*, 1864–1874.

[26] B. R. Miller III, T. D. McGee Jr, J. M. Swails, N. Homeyer, H. Gohlke, A. E. Roitberg, "MMPBSA. py: an efficient program for end-state free energy calculations" *J. Chem. Theory Comput.* 2012, *8*, 3314–3321.
